# Rare *MTNR1B* variants causing diminished MT2 signalling associate with elevated HbA_1c_ levels but not with type 2 diabetes

**DOI:** 10.1007/s00125-025-06381-y

**Published:** 2025-03-10

**Authors:** Kimmie V. Sørensen, Johanne M. Justesen, Lars Ängquist, Jette Bork-Jensen, Bolette Hartmann, Niklas R. Jørgensen, Jørgen Rungby, Henrik T. Sørensen, Allan Vaag, Jens S. Nielsen, Jens J. Holst, Oluf Pedersen, Allan Linneberg, Torben Hansen, Niels Grarup

**Affiliations:** 1 Novo Nordisk Foundation Center for Basic Metabolic Research, Faculty of Health and Medical Sciences, University of Copenhagen, Copenhagen, Denmark; 2 Department of Biomedical Sciences, Faculty of Health and Medical Sciences, University of Copenhagen, Copenhagen, Denmark; 3 Department of Clinical Biochemistry, Rigshospitalet, Glostrup, Denmark; 4 Department of Clinical Medicine, Faculty of Health and Medical Sciences, University of Copenhagen, Copenhagen, Denmark; 5 Translational Research Centre, Rigshospitalet, Copenhagen, Denmark; 6 Steno Diabetes Center Copenhagen, Herlev Hospital, Herlev, Denmark; 7 Department of Clinical Epidemiology, Aarhus University, Aarhus, Denmark; 8 Department of Epidemiology, Boston University, Boston, MA USA; 9 Lund University Diabetes Care, Lund University, Malmö, Sweden; 10 Department of Endocrinology, Skåne University Hospital, Malmö, Sweden; 11 Steno Diabetes Center Odense, Odense University Hospital, Odense, Denmark; 12 Department of Clinical Research, University of Southern Denmark, Odense, Denmark; 13 Department of Medicine, Faculty of Health and Medical Sciences, University of Copenhagen, Copenhagen, Denmark; 14 Center for Clinical Metabolic Research, Herlev-Gentofte Hospital, Copenhagen, Denmark; 15 Center for Clinical Research and Prevention, Copenhagen University Hospital-Bispebjerg and Frederiksberg, Copenhagen, Denmark

**Keywords:** Genetic association study, HbA_1c_, Melatonin, Melatonin receptor type 2, MT2, *MTNR1B*, Recall-by-genotype investigation, rs10830963, Type 2 diabetes, Variants impairing receptor function

## Abstract

**Aims/hypothesis:**

An intronic variant (rs10830963) in *MTNR1B* (encoding the melatonin receptor type 2 [MT2]) has been shown to strongly associate with impaired glucose regulation and elevated type 2 diabetes prevalence. However, *MTNR1B* missense variants have shown conflicting results on type 2 diabetes. Thus, we aimed to gain further insights into the impact of *MTNR1B* coding variants on type 2 diabetes prevalence and related phenotypes.

**Methods:**

We conducted a cross-sectional study, performing *MTNR1B* variant burden testing of glycaemic phenotypes (*N*=248,454, without diabetes), other cardiometabolic phenotypes (*N*=330,453) and type 2 diabetes prevalence (case–control study; *N*=263,739) in the UK Biobank. Similar burden testing with glycaemic phenotypes was performed in Danish Inter99 participants without diabetes (*N*=5711), and type 2 diabetes prevalence (DD2 cohort serving as cases [*N*=2930] and Inter99 serving as controls [*N*=4243]). Finally, we evaluated the effects of *MTNR1B* variants on the melatonin-induced glucose regulation response in a recall-by-genotype study of individuals without diabetes.

**Results:**

In the UK Biobank, *MTNR1B* variants were not associated with cardiometabolic phenotypes, including type 2 diabetes prevalence, except that carriers of missense *MTNR1B* variants causing impaired MT2 signalling exhibited higher HbA_1c_ levels compared with non-carriers (effect size, β, 0.087 SD [95% CI 0.039, 0.135]). Similarly, no significant associations were observed with phenotypes associated with glycaemic phenotypes in the Inter99 population. However, carriers of variants impairing MT2 signalling demonstrated a nominally significant lower glucose-stimulated insulin response (β −0.47 SD [95% CI −0.82, −0.11]). A reduced insulin response was also observed in carriers of variants impairing MT2 signalling (β −476.0 [95% CI −928.6, −24.4]) or the rs10830963 variant (β −390.8 [95% CI −740.1, −41.6]) compared with non-carriers after melatonin treatment.

**Conclusions/interpretation:**

The higher type 2 diabetes prevalence previously observed in carriers of missense *MTNR1B* variants causing impairment in MT2 signalling was not replicated in the UK Biobank, yet carriers had elevated HbA_1c_ levels.

**Data availability:**

Data (Inter99 cohort and recall-by-genotype study) are available on reasonable request from the corresponding author. Requests for DD2 data are through the application form at https://dd2.dk/forskning/ansoeg-om-data. Access to UK Biobank data can be requested through the UK Biobank website (https://www.ukbiobank.ac.uk/enable-your-research).

**Graphical Abstract:**

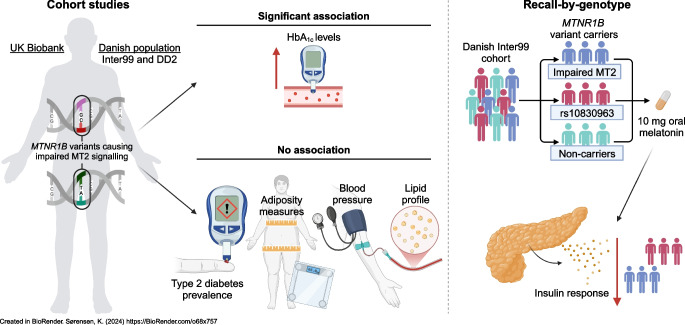

**Supplementary Information:**

The online version of this article (10.1007/s00125-025-06381-y) contains peer-reviewed but unedited supplementary material.


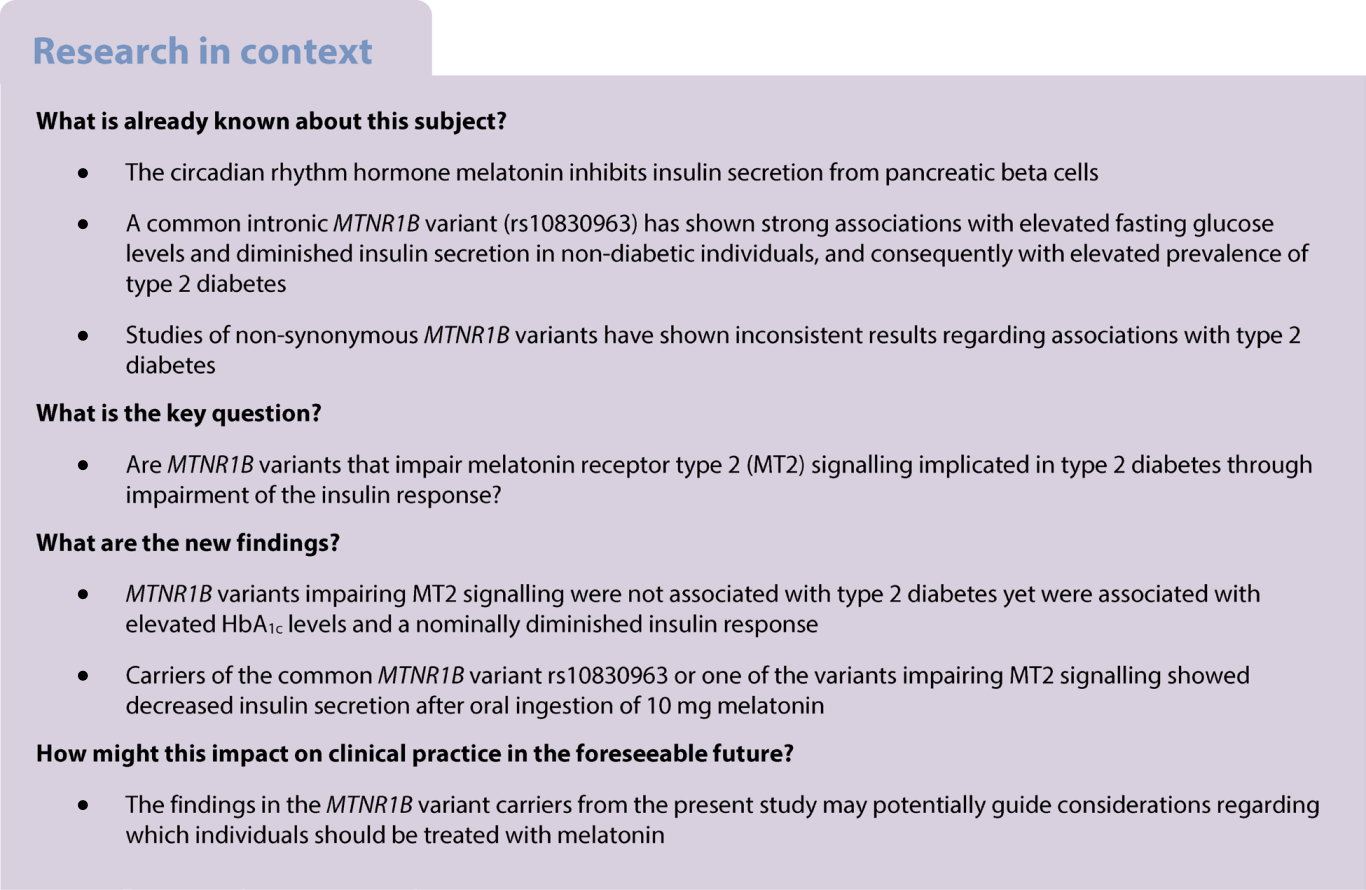


## Introduction

Manufactured melatonin is used globally to relieve sleep problems and jet lag, with its usage increasing the past decade even at doses above 5 mg/day [1]. Melatonin is a hormone constitutively secreted predominantly by the pineal gland, with plasma levels peaking at night because synthesis is inhibited by light exposure [2, 3]. To elicit its circadian functions, melatonin binds to G protein-coupled receptors (GPCRs) such as the melatonin receptor type 2 (MT2) on pancreatic beta cells [4–6], whereby rising circulating melatonin levels decrease insulin secretion [4, 7].

The implications of the melatonin–MT2 system in glucose homeostasis have been established through studies of genetic polymorphisms in *MTNR1B*, which encodes MT2. Carriage of a common intronic *MTNR1B* variant, rs10830963, has shown strong association with high fasting plasma glucose (FPG) levels [4, 8, 9], low beta cell glucose sensitivity [10], early phase insulin secretion [8, 10, 11], and a significantly greater prevalence of prediabetes [9] and type 2 diabetes than observed in non-carriers [8, 9, 12]. One study found that a common coding variant (G24E) was associated with elevated BMI and prevalence of obesity, but diminished FPG [13]. In this study, neither of six missense *MTNR1B* variants was associated with type 2 diabetes (*N*=8592 Danish individuals, 3617 with type 2 diabetes) [13]. However, another study identified 40 non-synonymous variants by sequencing, of which the combined burden of rare variants (minor allele frequency [MAF] <0.1%), particularly the variants impairing MT2 signalling, was associated with elevated type 2 diabetes prevalence (*N*=7632 European individuals, 2186 with type 2 diabetes) [14]. An understanding of how coding *MTNR1B* variants, particularly those impairing MT2 function, contribute to increased type 2 diabetes risk is still lacking but is crucial for gaining deeper insight into the role of the melatonin–MT2 system in the pathophysiology of type 2 diabetes.

Here, we aimed to gain further insight into the effects of *MTNR1B* variations on type 2 diabetes and type 2 diabetes endophenotypes by comprehensively examining coding *MTNR1B* variants through combining genetic association studies of quantitative traits from the UK Biobank and Danish cohorts, and through a recall-by-genotype investigation of selected *MTNR1B* variants.

## Methods

### Cross-sectional studies

#### Exome sequencing data from the UK Biobank

The UK Biobank is a prospective cohort study with a large catalogue of phenotypic and genotypic data from ~500,000 individuals (54% female; sex was acquired from central registry at recruitment or self-reported). Participants, aged 40–69 years at recruitment (2006–2010), were residents of the UK and represented a range of sociodemographic backgrounds, providing a comprehensive sample of the adult to elderly population. The study was approved by the North West Centre for Research Ethics Committee (11/NW/0382), and the participants provided signed consent before examination [15].

We used exome sequencing data from the UK Biobank (*N*=469,835) through application ID 32683 to identify *MTNR1B* variations. Quality control was performed in Hail (Hail Team. Hail 0.2.116. https://github.com/hail-is/hail). We performed additional quality control on the pVCF file comprising the *MTNR1B* locus on chromosome 11, filtering the single-nucleotide variations and performing subsequent filtering to include only unrelated white participants, as previously described [16], leaving 422 *MTNR1B* variations (electronic supplementary material [ESM] Table 1) and 330,453 participants for variant burden testing. In a previous study, non-synonymous *MTNR1B* variants were molecularly characterised to either impair Gi signalling (denoted impaired-function variants) or have wild-type (WT) MT2 Gi signalling (WT-like variants) [14]. Henceforth, molecular impaired-function and WT-like variants refer to this study. Among the 422 variants, ten impaired-function variants (W22L, A52T, L60R, A74T, P95L, R138C, R138H, R138L, R222H and I353T) and 19 WT-like variants were represented (ESM Fig. 1a, ESM Table 1).

To perform case–control analyses, we implemented the Eastwood algorithm [17] to identify prevalent cases of type 2 diabetes and identified 15,285 cases. Controls were remaining participants, excluding individuals with prevalent type 2 diabetes, reporting taking insulin (data fields no. 6153 and no. 6177) at any time, starting insulin within 1 year after diabetes diagnosis (no. 2986), being diagnosed with diabetes (no. 130708 and no. 2443), or who had HbA_1c_ levels above 42 mmol/mol (6.0%), leaving 278,775 individuals as a control group. For analysis of glycaemic phenotypes (HbA_1c_ and random glucose levels), similar filtering was applied but with a cutoff for HbA_1c_ levels of >48 mmol/mol (6.5%), leaving 284,835 individuals without diabetes. For analysis of other cardiometabolic traits, all 330,453 individuals were included without filtering.

#### Targeted sequencing data from two Danish study cohorts

As previously described [18, 19], the Danish population-based study cohort Inter99 (51.3% female; sex was reported at recruitment), initiated in 1999, includes participants aged between 30 and 60 years who were living in the south-western part of Copenhagen County at recruitment, thereby representing the adult population of the Danish society. For each participant, detailed biochemistry, anthropometrics and health/lifestyle questionnaire data at baseline were available. The study was approved by the local ethical committee (KA 98 155), and all participants provided written consent before participation [18, 19]. Targeted sequencing data were available for 6089 individuals, 5711 of whom did not have diabetes at baseline (4333 with normal glucose tolerance [NGT] and 1378 with prediabetes, according to WHO 1999 criteria [2 h plasma glucose 7.8–11.0 mmol/l and/or fasting plasma glucose 6.1–6.9 mmol/l]).

The Danish Centre for Strategic Research in Type 2 Diabetes (DD2) cohort is a large ongoing prospective type 2 diabetes research study, initiated in 2010, that, on a continuous basis, enrols individuals recently diagnosed with type 2 diabetes. DD2 was approved by the ethical committee of the Region of Southern Denmark and by the Danish National Committee on Biomedical Research Ethics, and all participants provided signed consent before participation [20]. Targeted sequencing data was available for 2930 participants (age 21–95 years, 40% female).

Targeted sequencing of *MTNR1B* was performed, as previously described [16]. The *MTNR1B* locus reached a minimum per-base mean depth of 68× and a median per-base mean coverage for the target region of 225×. We lifted the dataset to GRCh38 by using LiftOver before variant annotation, revealing 76 *MTNR1B* variations (ESM Table 2). Of the molecularly characterised variants [14], 11 were represented, of which five (A52T, L60R, R138C, R222H, and I353T) were impaired-function variants (ESM Fig. 1b).

To perform case–control analyses of type 2 diabetes in the Danish population, the DD2 cohort provided type 2 diabetes cases (*N*=2930) and Inter99 provided control individuals with NGT based on fasting glucose (<6.1 mmol/l) and 2 h OGTT glucose (<7.8 mmol/l) levels at baseline and no diabetes diagnosis according to registry data until 2017 (*N*=4243). For analysis of quantitative traits, only Inter99 individuals without diabetes at baseline were included (*N*=5711).

#### Variant annotation

We used the exon locations (https://genome.ucsc.edu/cgi-bin/hgTables) of *MTNR1B* (NM_005959.3) ±50 bp overhangs to extract variants for annotation [21, 22]. We subjected the coding *MTNR1B* variants (transcript ENST00000257068) to 17 dbNSFP4 variant effect predictors aggregated into four masks [23]. A variant was predicted to cause loss of function (pLoF variant) if it passed at least one mask: mask (1) LOFTEE = HC; mask (2) VEST4 rankscore >0.9, CADD rankscore >0.9, DANN rankscore >0.9, Eigen-raw rankscore >0.9 and Eigen-PC-raw rankscore >0.9; mask (3) FATHMM prediction = D, FATHMM-MKL prediction = D, PROVEAN prediction = D, MetaSVM prediction = D, MetaLR prediction = D, and MCAP score >0.025; or mask (4) PolyPhen HDIV prediction = D, PolyPhen HVAR prediction = D, SIFT prediction = D, LRT prediction = D, MutTaster prediction = D or A. In the UK Biobank, 77 pLoF variants were identified (ESM Table 3), comprising four impaired-function variants (P95L, R138C, R138H and R138L) and five WT-like variants (G109A, S123R, V124I, T201M and R316H). In the Danish cohorts, nine variants were pLoF variants (ESM Table 4), including the impaired-function variant R138C and the WT-like variant V124I.

#### Outcome measures

Outcomes included changes in quantitative glycaemic phenotypes (HbA_1c_ and random glucose levels in the UK Biobank, levels of fasting glucose and insulin, OGTT glucose and insulin and HbA_1c_ as well as insulin secretion and sensitivity indexes in the Danish Inter99 cohort), other cardiometabolic phenotypes (adiposity measures, BP and lipid profile) and type 2 diabetes prevalence. Indexes of insulin secretion and sensitivity were calculated as follows: corrected insulin response (CIR) [24]; beta cell function insulin sensitivity (BIGTT)–acute insulin response (BIGTT-AIR) and insulin sensitivity index (BIGTT-SI) [25]; HOMA-IR [26]; and Stumvoll insulin sensitivity index [27].

#### Variant burden testing

We collapsed coding variants into groups according to functional annotation or previous molecular phenotype for burden testing, in addition to linear (quantitative traits) and logistic (case–control) regression: synonymous variants; WT-like variants excluding the common G24E variant (MAF=0.09); the G24E variant; missense variants excluding WT-like variants; impaired-function variants; and pLoF variants excluding WT-like variants. The analyses of UK Biobank data were conducted on its Research Analysis Platform (https://ukbiobank.dnanexus.com). We used the R package *SKAT* [28] for burden testing i.e. SKAT (‘r.corr=1’) with equal weights for molecularly tested variants, and SKAT-optimal [29] (‘method = optimal.adj’) for uncharacterised variants. We used R statistical software (v.4.3.3 for Danish cohort analysis, and v.4.2.2 for UK Biobank analysis) [30] to perform the association tests, adjusted for sex, age, age^2^ and four (Danish cohort analyses) or 20 (UK Biobank analyses) genetic principal components (accounting for population stratification [ethnicity]), as well as BMI, where described. The quantitative phenotypes were inverse rank-normalised before analysis: β (effect size) was represented as SD. Samples with missing phenotypes or covariates were excluded from corresponding analysis, while imputation was applied for missing genotypes. OR for the case–control studies was calculated as the exponential of the β, deriving from the logistic regression model. Visualisation of the results was performed with the R package *metafor* [31]. Bonferroni correction for multiple testing led to a significance threshold of 3.6 × 10^−3^ (14 tests) and 3.8 × 10^−3^ (13 tests) for analysis in the UK Biobank and the Danish cohorts, respectively.

#### Statistical power considerations

The least detectable effect (LDE) of a two-sided *t* test for quantitative phenotypes (standardised) was calculated for comparison between groups of unequal size. Depending on the number of carriers, the LDEs to achieve 80% power ranged from 0.018 to 0.077 SD and 0.13 to 0.66 SD for the analyses in the UK Biobank and the Danish population, respectively. For the type 2 diabetes analyses, the required LDE ORs ranged from 1.09 to 1.39 and 1.63 to 5.04 for the UK Biobank and the Danish cohorts, respectively, to achieve 80% power (assuming the non-carrier prevalence to be 5%).

### Recall-by-genotype investigation

#### Study design

We conducted a recall-by-genotype investigation (approved by the Ethical Committee of the Capital Region of Denmark [H-17023209]). We recruited individuals from the Inter99 cohort according to their *MTNR1B* genotype: (1) carriers of impaired-function variants [14] (A52T, L60R, R138C, R222H and I353T), according to targeted sequencing data; (2) homozygous carriers of the intronic variant (rs10830963), according to genotyping (method as previously described [32]); and (3) non-carriers of these six variants. To have 80% power to detect an effect of 0.91 SD (for two-tailed unpaired *t* tests and a statistical significance threshold of 0.05), we aimed for 20 individuals in each group. Individuals in groups 2 and 3 were matched to group 1 (three matches per variant carrier) by age (±1.5 years), sex and BMI (±1.5 kg/m^2^). Exclusion criteria included diabetes, kidney dysfunction, liver disease, sleep disorder, regular intake of melatonin, current or prior alcohol or drug use, use of antidepressant or antipsychotic medication, shift work or melatonin allergy. All participants provided written informed consent, and 36 completed the study. No differences in age and BMI were found across groups (*p*>0.05).

Participants completed two examination days (~7 days apart) after an overnight fast. Blood samples were drawn from a catheter placed in an antecubital vein. On visit 1, they underwent BP (automated sphygmomanometer) and anthropometric measurements (bio-electrical impedance) as well as a 2 h 75 g OGTT with 100 ml water, with blood samples drawn at −5, 0, 15, 30, 45, 60, 90 and 120 min. On visit 2, participants ingested a 10 mg melatonin capsule 50 min before a 2 h 75 g OGTT, with blood samples drawn at −60, −55, −5, 0, 15, 30, 45, 60, 90 and 120 min. Participants remained awake throughout the examinations. Data were collected from May 2018 until December 2018.

#### Outcome measures

Plasma glucose was measured on the VITROS 4600/5600 automated analyser (QuidelOrtho, San Diego, CA) using the VITROS Chemistry Products Glu Slides (Quidel Ortho). Insulin was measured on the Liaison XL immune assay analyser (DiaSorin, Saluggia, Italy) using the Liaison Insulin assay (DiaSorin). Plasma total glucose-dependent insulinotropic polypeptide (GIP) and total glucagon-like peptide-1 (GLP-1) were measured with sandwich ELISA kits (catalogue no. 10–1258-01 and 10–1278-01, Mercodia, Uppsala, Sweden); detection limits were 1.62 pmol/l and 0.65 pmol/l, respectively. Plasma was extracted in a final concentration of 70% vol./vol. ethanol, followed by glucagon measurement using a C-terminally directed RIA (antiserum code no. 4305), measuring glucagon of pancreatic origin as previously described [33]; sensitivity was below 1 pmol/l and intra assay CV below 10%. Serum melatonin was measured with an RIA kit (catalogue no. RE29301; IBL, Hamburg, Germany; detection limit 0.9 pg/ml). AUC values were calculated using the trapezoidal rule.

#### Statistical comparisons

Using R (v.4.3.3) [30], we compared values at visit 1 (glucose stimulation) with those at visit 2 (glucose and melatonin stimulation) by paired *t* tests to consider melatonin-induced differences. To judge significance of pairwise group differences, unpaired *t* tests were performed. If the *t* test model assumptions were not met, we used the non-parametric Wilcoxon signed-rank test. Difference-in-differences were considered by subtracting the value at visit 1 from that at visit 2. We used a statistical significance threshold of 0.05, with no correction for multiple testing.

## Results

### *MTNR1B* variant group-based association analysis in the UK Biobank

**Fig. 1:**
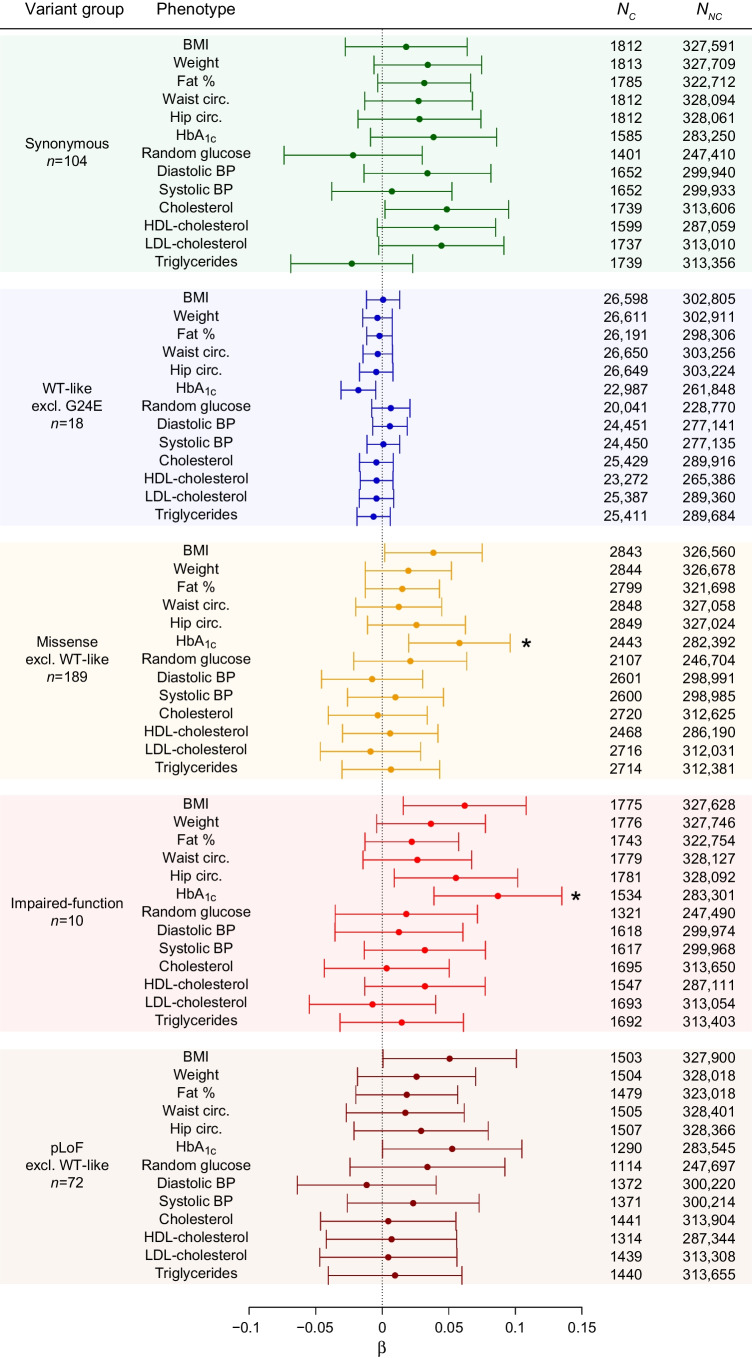
Group-based burden testing of cardiometabolic phenotypes in the UK Biobank. The forest plot shows β (as SD, with error bars representing the 95% CI) for each association analysis between the variant groups and glycaemic phenotypes (*N*=284,835) or other cardiometabolic phenotypes (*N*=330,453). *p<3.6 × 10^−3^ (association reached corrected significance threshold). circ., circumference; excl., excluding; *n*, no. of variants in the variant group;* N*_*C*_, no. of carriers; *N*_*NC*_, no. of non-carriers

Group-based variant burden tests were performed for cardiometabolic phenotypes (*N*=330,453), glycaemic phenotypes (*N*=284,835) and type 2 diabetes prevalence (*N*=294,060). None of the variant groups, nor the G24E variant, was associated with type 2 diabetes, adiposity measures or the lipid profile (Table 1, Fig. 1 and ESM Fig. 2a, ESM Table 5). The groups of missense variants and impaired-function variants showed associations with elevated HbA_1c_ levels (missense variants, β 0.058 SD [95% CI 0.020, 0.096], *p*=2.9 × 10^−3^; impaired-function variants, β 0.087 SD [95% CI 0.039, 0.135], *p*=5.6 × 10^−4^) after correction for multiple testing (Fig. 1 and ESM Fig. 2a, ESM Table 5). Combining pLoF and impaired-function variants did not add further phenotype associations (ESM Fig. 2b, ESM Table 5). Similar observations were made after adjustment for BMI (data not shown), though the association between the missense variants and HbA_1c_ levels did not remain statistically significant (*p*=7.7 × 10^−3^).

**Table 1:** Group-based burden testing for type 2 diabetes prevalence in the UK Biobank (*N*=294,060)

Variant group	T2D cases		Controls		OR (95% CI)	*p* value
	Carriers	Non-carriers	Carriers	Non-carriers		
Synonymous	66	15,219	1542	277,233	0.76 (0.59, 0.97)	0.040
WT-like, excluding G24E	1167	14,118	22,500	256,275	0.94 (0.88, 1.00)	0.081
Missense, excluding WT-like	136	15,149	2394	276,381	1.04 (0.87, 1.24)	0.86
Impaired-function variants	81	15,204	1505	277,270	0.98 (0.78, 1.23)	0.83
pLoF, excluding WT-like	75	15,210	1267	277,508	1.08 (0.85, 1.37)	0.70

*p* values are derived from burden testing (SKAT or SKAT-O)

T2D, type 2 diabetes

### Burden testing of glycaemic phenotypes in the Danish population

**Fig. 2:**
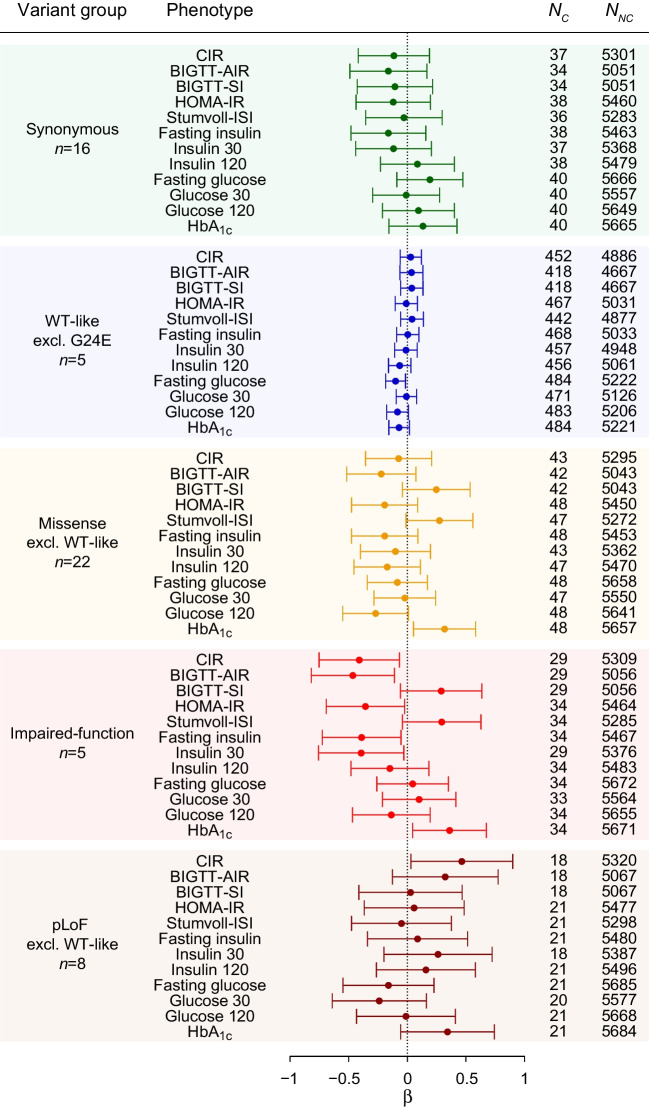
Variant group-based associations with glycaemic phenotypes in participants without diabetes in the Danish Inter99 cohort (*N*=5711). The forest plot shows β (as SD, with error bars representing the 95% CI) for each group-based association analysis. Time points of 30 and 120 represent minutes after OGTT initiation. None of the tests reached the corrected significance threshold (*p*<3.8 × 10^−3^). CIR, corrected insulin response index; excl. excluding; ISI, insulin sensitivity index;* n*, no. of variants in the variant group; *N*_*C*_, no. of variant carriers in the given variant group; *N*_*NC*_, no. of non-carriers

Because the impaired-function variants were associated with elevated HbA_1c_, we performed burden testing in the Danish cohorts with detailed phenotypes for studying glucose regulation in participants without diabetes (Inter99; *N*=5711), and type 2 diabetes in clinically diagnosed cases (*N*=2930) and OGTT-determined NGT controls (*N*=4243). None of the variant groups was significantly associated with the glycaemic phenotypes or type 2 diabetes prevalence after correction for multiple testing (Fig. 2 and ESM Tables 6, 7), except for the common WT-like G24E variant displaying an elevated estimated insulin response (*p*=0.0032; ESM Fig. 3a, ESM Table 6) (after BMI adjustment, *p*=0.024). However, in line with the findings in the UK Biobank, the impaired-function variant carriers displayed nominally diminished estimated insulin response (β −0.47 SD [95% CI −0.82, −0.11]) and nominally elevated HbA_1c_ levels (β 0.360 SD [95% CI 0.046, 0.675]) compared with non-carriers (Fig. 2 and ESM Table 6). Neither combining pLoF and impaired-function variants (ESM Fig. 3b, ESM Table 6) nor adjustment for BMI (data not shown) changed these interpretations.

### Recall-by-genotype investigation

#### Baseline characteristics of participants

**Fig. 3:**
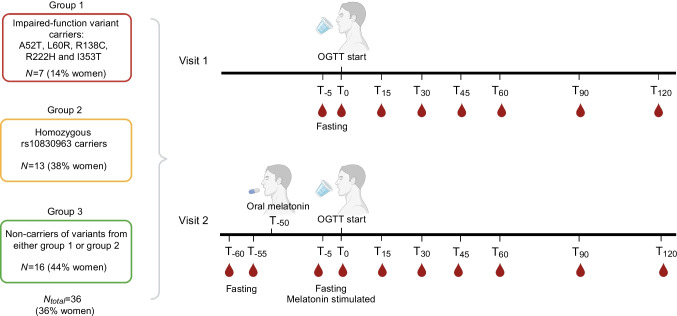
Workflow of the recall-by-genotype investigation. Participants were recruited from the Danish Inter99 cohort according to *MTNR1B* variants: group 1, individuals carrying one of the impaired-function *MTNR1B* variants (A52T, L60R, R138C, R222H and I353T); group 2, homozygous carriers of the common *MTNR1B* rs10830963 variant; and group 3, individuals not carrying any of the six *MTNR1B* variants. In the impaired-function *MTNR1B* variant group, one carried A52T, one carried L60R, two carried R138C and three carried I353T. No individuals carrying the R222H variant were able to participate in the study. Each participant completed two examination days separated by ~7 days. Created in BioRender. Sørensen, K. (2024) BioRender.com/x64 g373. T, time (min)

To obtain a better physiological understanding of impaired MT2 signalling in glucose regulation, we conducted a recall-by-genotype study (Fig. 3). Participants underwent two examinations: a 2 h OGTT at visit 1 (referred to as glucose stimulation); and 10 mg oral melatonin administered 50 min before the OGTT at visit 2 (referred to as glucose and melatonin stimulation). The participants’ baseline characteristics are provided in ESM Table 8.

#### Melatonin-induced altered glucose regulation

**Fig. 4:**
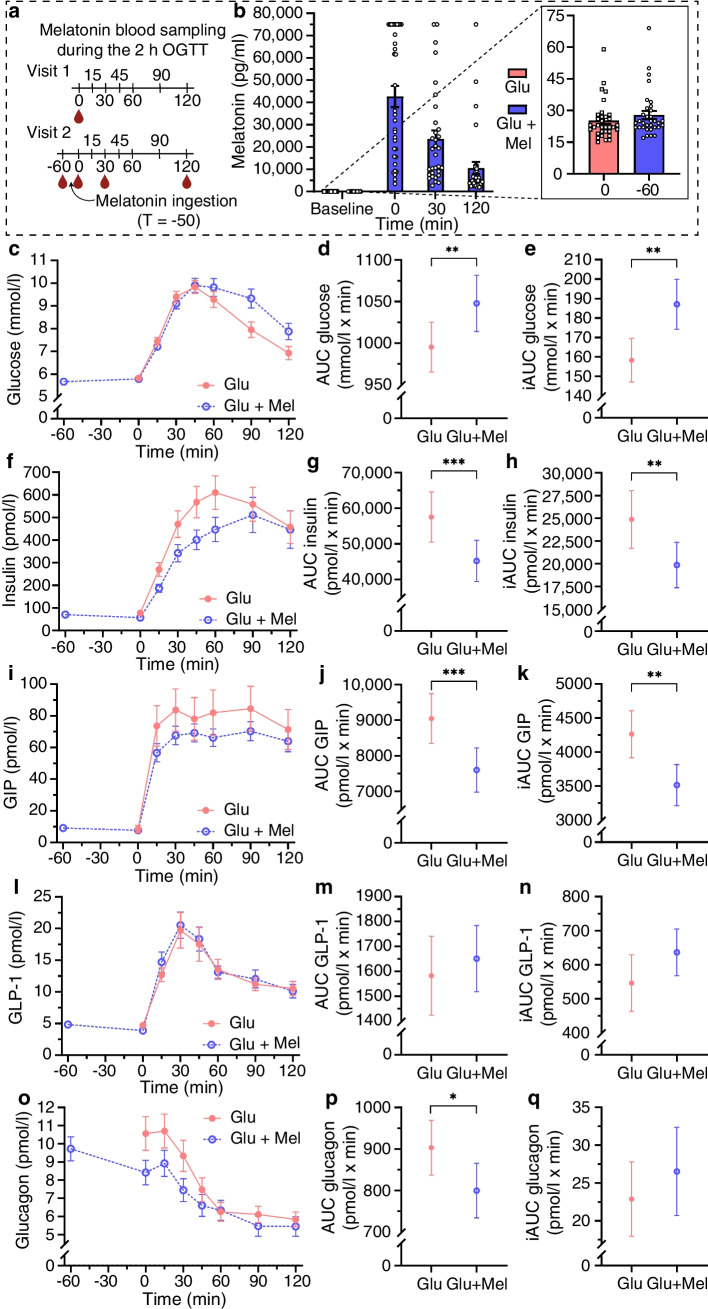
Acute effects of melatonin stimulation on circulating glucose, insulin, GIP, GLP-1 and glucagon levels after a glucose load in combined genetic groups. The changes in levels during a 2 h OGTT are shown. AUC and iAUC were calculated from the OGTT data. The OGTT stimulated with only glucose is denoted ‘Glu’, whereas the OGTT stimulated with both glucose and melatonin is denoted ‘Glu + Mel’. (**a**) Blood sample drawn to measure circulating melatonin during OGTT. The blood drop was created with Biorender.com. (**b**) Circulating levels of melatonin during OGTT. (**c**–**e**) Glucose levels (**c**), AUC (**d**) and iAUC (**e**) during OGTT. (**f**–**h**) Insulin levels (**f**), AUC (**g**) and iAUC (**h**) during OGTT. (**i**–**k**) GIP levels (**i**), AUC (**j**) and iAUC (**k**) during OGTT. (**l**–**n**) GLP-1 levels (**l**), AUC (**m**) and iAUC (**n**) during OGTT. (**o**–**q**) Glucagon levels (**o**), AUC (**p**) and iAUC (**q**) during OGTT. The data represent mean ± SEM. *p<0.05, **p<0.01, ***p<0.001. Glu, glucose; Mel, melatonin; T, time (min)

We investigated melatonin’s effects by combining all genotype groups (*N*=36). During melatonin-stimulated OGTTs (Fig. 4a), circulating melatonin showed a ~1500-fold increase from baseline to OGTT initiation (time 0 min) (Fig. 4b). The FPG levels were lower at visit 2 before melatonin administration than at visit 1 (estimated effect [β] −0.157 [95% CI −0.255, −0.059] mmol/l, *p*=0.004). No differences in circulating fasting insulin, GIP, GLP-1 or glucagon levels between visits (before melatonin administration) were observed (data not shown).

Melatonin and glucose stimulation, compared with glucose stimulation alone, increased glucose levels after OGTT at 60 min (Fig. 4c) and resulted in a higher AUC_glucose_ (β 66.9 [95% CI 25.4, 108.3] mmol/l × min, *p*=0.002) and incremental AUC (iAUC) of glucose (β 33.1 [95% CI 12.2, 54.1] mmol/l × min, *p*=0.003) (Fig. 4d, e). Melatonin decreased insulin levels within the first 60 min (Fig. 4f), as evidenced by a lower AUC_insulin_ (β −10,391 [95% CI −17,895, −2886] pmol/l × min, *p*=0.0004) and iAUC_insulin_ (β −4183 [95% CI −7706, −660] pmol/l × min, *p*=0.010) (Fig. 4g, h), and impaired the insulin response, as indicated by decreased CIR, BIGTT-AIR and insulin AUC during 0–30 min (Table 2). However, melatonin increased BIGTT-SI and decreased HOMA-IR, thereby suggesting increased insulin sensitivity (Table 2). Melatonin decreased circulating GIP (AUC_GIP_, β −1530 [95% CI −2381, −680] pmol/l × min, *p*=0.0009; iAUC_GIP_, β −780 [95% CI −1256, −304] pmol/l × min, *p*=0.002) (Fig. 4i–k) but not GLP-1 (Fig. 4l–n and ESM Table 9), and induced lower glucagon levels up to 30 min in the OGTT (Fig. 4o–q and ESM Table 9).

**Table 2:** Effects of melatonin on surrogate measures of insulin secretion and sensitivity

Measurement	Glucose stimulation (visit 1)	Glucose + melatonin stimulation (visit 2)	Estimated effect (95% CI)	*p* value
CIR	932.8 ± 108.5	738.4 ± 76.9	−194.4 (−341.4, −47.3)	0.0074
BIGTT-AIR	2506.7 ± 484.9	1874.3 ± 168.9	−657.0 (−1408.2, 94.2)	0.048
AUC30_insulin_ (pmol/l × min)	8155 ± 947	5793 ± 578	−2363 (−3467, −1258)	1.6 × 10^−6^
BIGTT-SI	6.39 ± 0.75	7.04 ± 0.77	0.64 (0.13, 1.14)	0.016
HOMA-IR	3.46 ± 0.43	2.55 ± 0.45	−0.93 (−1.51, −0.34)	9.7 × 10^−6^

Data are presented as mean ± SE

AUC30, AUC at 0–30 min in OGTT

#### Acute effects of melatonin in *MTNR1B* variant carriers

**Fig. 5:**
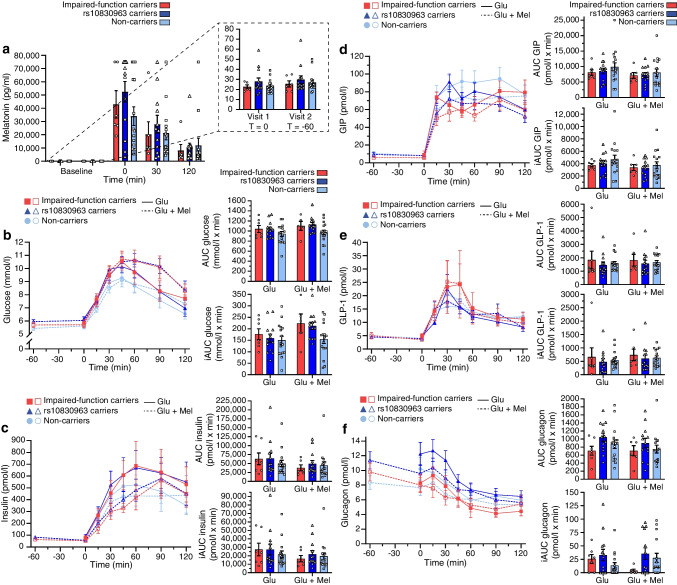
Effects of melatonin on biochemical measures, stratified by genetic group. The following genetic groups are shown: impaired-function *MTNR1B* variant carriers (red); rs10830963 carriers (dark blue); and non-carriers (light blue). (**a**) Circulating levels of melatonin before stimulation with glucose (visit 1) or glucose and melatonin (visit 2), and during OGTT with stimulation with melatonin (times 0, 30 and 120 min). (**b**–**f**) Circulating glucose (**b**), insulin (**c**), GIP (**d**), GLP-1 (**e**) and glucagon levels (**f**) after stimulation with glucose only or with glucose and melatonin, with corresponding AUC and iAUC measures shown. All data represent mean ± SEM. Glu, glucose; Mel, melatonin; T, time (min)

As our main hypothesis was that impaired-function variant carriers had an altered insulin response, we compared melatonin’s acute effects and the phenotypic changes after glucose stimulation vs melatonin and glucose stimulation (difference-in-differences) between groups. Melatonin levels were comparable among genetic groups (Fig. 5a). After melatonin administration, both impaired-function variant carriers and rs10830963 variant carriers exhibited a lower CIR index than non-carriers (impaired-function variants, β −476.0 [95% CI −927.6, −24.4], *p*=0.033; rs10830963 variant, β −390.8 [95% CI −740.1, −41.6], *p*=0.028), conjointly with higher glucose levels (Fig. 5b) in rs10830963 variant carriers only (iAUC_glucose_ *p*<0.027; ESM Table 10). Carriage of an *MTNR1B* variant had no influence on the melatonin-induced response on insulin, GIP, GLP-1 and glucagon levels (Fig. 5c–f, ESM Tables 10, 11).

## Discussion

Understanding genetic variations in GPCR signalling enhances our knowledge of receptor function and disease pathophysiology [34–36]. We comprehensively investigated MT2 coding variants because of previous associations of *MTNR1B* rs10830963 and impaired-function variants with type 2 diabetes risk [8–11, 14]. In summary, we did not find missense or impaired-function variants to be associated with type 2 diabetes. However, we demonstrated that missense *MTNR1B* variants, particularly impaired-function variants, associated with elevated HbA_1c_ levels and nominally diminished insulin response, supported by a recall-by-genotype study showing indications of poor glucose regulation in carriers of impaired-function or rs10830963 variants after melatonin ingestion.

The unexpected lack of association between the impaired-function variants and elevated type 2 diabetes prevalence contradicts the findings of a previous study [14]. However, the LDE ranged from OR 1.09 to OR 1.39 (from highest to lowest numbers of carriers) in the UK Biobank, with low numbers of carriers of impaired-function variants; this may have limited the detectability of small effects. Additionally, reliance on self-reported data for type 2 diabetes classification [17] may have misclassified cases as controls. Furthermore, the participants may be healthier than the general population, potentially skewing disease prevalence estimates [37].

The WT-like G24E variant has been reported to associate with diminished FPG and elevated insulinogenic index and BMI [13]. We replicated only a higher insulin response, an effect opposite to the nominal findings of the impaired-function variants. Despite elevated cell surface expression, previous studies [13, 14, 38] found no evidence of enhanced signalling to explain this opposing effect direction.

The impaired-function variants molecularly impair MT2 signalling [14]; hence, both impaired MT2 signalling and enhanced *MTNR1B* expression (potentially enhancing signalling), as observed for the rs10830963 variant [4, 39], may increase glucose levels, potentially through a diminished insulin response. However, the impaired-function variant carriers showed only a nominally reduced insulin response compared with non-carriers in the Danish population. This result was supported by the diminished insulin response in these carriers following melatonin administration. Prior functional studies of *MTNR1B* variants have focused on Gi, Gz, cAMP production, ERK activation and β-arrestin recruitment [14, 38]. Thus, other downstream pathways could be altered by the impaired-function variants, thereby explaining why enhanced *MTNR1B* expression and impaired MT2 signalling showed comparable physiological outcomes. The melatonin-induced inhibition of insulin secretion observed by us and others [4, 7], particularly at night, may be a mechanism preventing beta cell exhaustion and enhancing survival. The increased apoptosis observed in pancreatic beta cells after hyperglycaemia or hyperlipidaemia has been shown to be attenuated after melatonin administration potentially through its antioxidant properties [40, 41]. Testing whether *MTNR1B* impaired-function variants cause dysfunctional protection against beta cell apoptosis may provide insights into whether MT2 signalling also contributes to beta cell survival, thereby influencing glucose regulation associated with these variants.

The observed melatonin-induced effects on the insulin response and glucose levels align with results from a study of 3 months of treatment with 4 mg of melatonin at bedtime in non-diabetic individuals, showing decreased first-phase insulin secretion and elevated glucose levels, particularly in homozygous rs10830963 carriers [39]. However, further investigation is required to determine whether similar long-term melatonin-induced effects occur in carriers of impaired-function variants. The observed lower glucagon release was not reported as an acute effect in 20 healthy men nor as a long-term effect in 17 individuals with type 2 diabetes receiving 10 mg melatonin [42, 43]. Nonetheless, the effects on glucagon levels could also result from activation of the melatonin receptor type 1, with presumed expression in pancreatic alpha cells [44]. Similar melatonin-induced lower GIP levels were observed in 15 healthy men, with preserved incretin actions [45]. Thus, melatonin-mediated lower GIP levels may not cause the impaired glucose regulation. Yet, decreased insulin sensitivity and increased insulin secretion have been observed in individuals with type 2 diabetes treated with 10 mg melatonin at bedtime for 3 months [42]. Furthermore, melatonin has been shown to decrease oxidative stress (potentially through its antioxidant molecular structure [46]), improve metabolic profiles in individuals with type 2 diabetes and CHD [47], and lower diurnal BP [48]. Furthermore, a meta-analysis of 16 RCTs has reported melatonin-induced decreases in FPG, HbA_1c_ (mean difference of −0.38% (4.2 mmol/mol) [95% CI −0.67, −0.10]) and insulin resistance [49]. Hence, controversial effects of melatonin treatment have been reported in individuals with type 2 diabetes and in healthy individuals.

Studying rare genetic variants presents several limitations, particularly due to low numbers of carriers, which reduces the power to detect small effect sizes. Based on the LDEs, the analyses in the UK Biobank were expected to detect small effects. Thus, the elevated HbA_1c_ levels observed in carriers of impaired-function variants that survived correction for multiple testing are unlikely to be false-positives, whereas non-significant results may be interpreted as negative findings. However, correcting for multiple testing of related phenotypes increases the risk of true effects becoming false-negatives. The Danish cohort analyses were therefore interpreted cautiously due to the moderate effect size required for detectability based on the LDEs. These analyses showed only nominal associations, such as between impaired-function variants and a diminished insulin response and elevated HbA_1c_ levels, but lacked the statistical power to survive correction for multiple testing. The limited number of rare variant carriers was particularly evident in the recall-by-genotype study. Despite significant efforts, only seven impaired-function variant carriers from the Inter99 cohort completed the study, far below the 20 required in each group for predefined 80% statistical power. Consequently, the findings must be interpreted cautiously. The limited statistical power also constrained the conduction of sex-stratified analyses. However, we accounted for sex-derived differences in our regression models (i.e. sex-derived differences do not influence the interpretation of our results). Another limitation of the recall-by-genotype study was the lack of correction for multiple testing. However, given the pre-specified main hypothesis that carriers of impaired-function variants exhibit altered insulin response to melatonin treatment compared with non-carriers, maintaining a significance threshold of 0.05 for this test may be justifiable. Secondary analyses, however, should be interpreted with extra caution. Nevertheless, it is notable that our observations across cohorts and studies demonstrated consistent effect directions aligning with logical phenotypic associations; elevated HbA_1c_ levels coincided with a nominally diminished insulin response. Future larger studies should investigate the impaired-function variants further in relation to detailed glucose-regulation phenotypes to confirm or refute this study’s indications that the increased HbA_1c_ levels may be linked to diminished insulin responses.

In conclusion, our findings suggest that carriers of *MTNR1B* variants impairing MT2 signalling have elevated HbA_1c_ levels and potentially diminished insulin responses. However, they have no significant change in type 2 diabetes prevalence, which contrasts with previous findings.

## Supplementary Information

Below is the link to the electronic supplementary material.

Supplementary file1 (XLSX 99.4 KB)

Supplementary file2 (PDF 1024 KB)

## Data Availability

Data (Inter99 cohort and recall-by-genotype study) are available on reasonable request from the corresponding author Niels Grarup (niels.grarup@sund.ku.dk) or Torben Hansen (torben.hansen@sund.ku.dk). Requests for DD2 data should be addressed to Kurt Højlund (kurt.hoejlund@rsyd.dk) and Jens S. Nielsen (jsn@rsyd.dk) through the application form at https://dd2.dk/forskning/ansoeg-om-data. Access to UK Biobank data can be requested through the UK Biobank website (https://www.ukbiobank.ac.uk/enable-your-research).

## Abbreviations

BIGTT-AIR: Beta cell function insulin sensitivity GTT–acute insulin response
BIGTT-SI: Beta cell function insulin sensitivity GTT–insulin sensitivity index
CIR: Corrected insulin response
DD2: Danish Centre for Strategic Research in Type 2 Diabetes
FPG: Fasting plasma glucose
GIP: Glucose-dependent insulinotropic polypeptide
GLP-1: Glucagon-like peptide-1
GPCR: G protein-coupled receptor
iAUC: Incremental AUC
LDE: Least detectable effect
MAF: Minor allele frequency
MT2: Melatonin receptor type 2
NGT: Normal glucose tolerance
pLoF: Predicted loss of function
WT: Wild-type

## References

[1] Li J, Somers VK, Xu H, Lopez-Jimenez F, Covassin N (2022) Trends in use of melatonin supplements among US adults, 1999–2018. JAMA 327(5):483–485. 10.1001/jama.2021.23652 35103775 10.1001/jama.2021.23652 PMC8808329

[2] Arendt J (1998) Melatonin and the pineal gland: influence on mammalian seasonal and circadian physiology. Rev Reprod 3(1):13–22. 10.1530/revreprod/3.1.13 9509985 10.1530/ror.0.0030013

[3] Anderson GM, Young JG, Cohen DJ, Young SN (1982) Determination of indoles in human and rat pineal. J Chromatogr 228:155–63. 10.1016/S0378-4347(00)80428-4 6176588 10.1016/s0378-4347(00)80428-4

[4] Lyssenko V, Nagorny CLF, Erdos MR et al (2009) A common variant in MTNR1B associated with increased risk of type 2 diabetes and impaired early insulin secretion. Nat Genet 41(1):82–88. 10.1038/ng.288 19060908 10.1038/ng.288 PMC3725650

[5] Peschke E, Stumpf I, Bazwinsky I, Litvak L, Dralle H, Mühlbauer E (2007) Melatonin and type 2 diabetes - a possible link? J Pineal Res 42(4):350–358. 10.1111/j.1600-079X.2007.00426.x 17439551 10.1111/j.1600-079X.2007.00426.x

[6] Nagorny CLF, Sathanoori R, Voss U, Mulder H, Wierup N (2011) Distribution of melatonin receptors in murine pancreatic islets. J Pineal Res 50(4):412–417. 10.1111/j.1600-079X.2011.00859.x 21355877 10.1111/j.1600-079X.2011.00859.x

[7] Peschke E, Peschke D, Hammer T, Csernus V (1997) Influence of melatonin and serotonin on glucose-stimulated insulin release from perifused rat pancreatic islets in vitro. J Pineal Res 23(3):156–163. 10.1111/j.1600-079X.1997.tb00349.x 9406987 10.1111/j.1600-079x.1997.tb00349.x

[8] Prokopenko I, Langenberg C, Florez JC et al (2009) Variants in MTNR1B influence fasting glucose levels. Nat Genet 41(1):77–81. 10.1038/ng.290 19060907 10.1038/ng.290 PMC2682768

[9] Sparsø T, Bonnefond A, Andersson E et al (2009) G-allele of intronic rs10830963 in MTNR1B confers increased risk of impaired fasting glycemia and type 2 diabetes through an impaired glucose-stimulated insulin release: studies involving 19,605 Europeans. Diabetes 58(6):1450–1456. 10.2337/db08-1660 19324940 10.2337/db08-1660 PMC2682679

[10] Langenberg C, Pascoe L, Mari A et al (2009) Common genetic variation in the melatonin receptor 1B gene (MTNR1B) is associated with decreased early-phase insulin response. Diabetologia 52(8):1537–1542. 10.1007/s00125-009-1392-x 19455304 10.1007/s00125-009-1392-x PMC2709880

[11] Staiger H, Machicao F, Schäfer SA et al (2008) Polymorphisms within the novel type 2 diabetes risk locus MTNR1B determine β-cell function. PLoS One 3(12):e3962. 10.1371/journal.pone.0003962 19088850 10.1371/journal.pone.0003962 PMC2597741

[12] Tan X, Ciuculete D-M, Schiöth HB, Benedict C (2020) Associations between chronotype, MTNR1B genotype and risk of type 2 diabetes in UK Biobank. J Intern Med 287(2):189–196. 10.1111/joim.12994 31623012 10.1111/joim.12994 PMC7003850

[13] Andersson EA, Holst B, Sparsø T et al (2010) MTNR1B G24E variant associates with BMI and fasting plasma glucose in the general population in studies of 22,142 Europeans. Diabetes 59(6):1539–1548. 10.2337/db09-1757 20200315 10.2337/db09-1757 PMC2874716

[14] Bonnefond A, Clément N, Fawcett K et al (2012) Rare MTNR1B variants impairing melatonin receptor 1B function contribute to type 2 diabetes. Nat Genet 44(3):297–301. 10.1038/ng.1053 22286214 10.1038/ng.1053 PMC3773908

[15] Bycroft C, Freeman C, Petkova D et al (2018) The UK Biobank resource with deep phenotyping and genomic data. Nature 562(7726):203–209. 10.1038/s41586-018-0579-z 30305743 10.1038/s41586-018-0579-z PMC6786975

[16] Melchiorsen JU, Sørensen KV, Bork-Jensen J et al (2023) Rare heterozygous loss-of-function variants in the human GLP-1 receptor are not associated with cardiometabolic phenotypes. J Clin Endocrinol Metab 108(11):2821–2833. 10.1210/clinem/dgad290 37235780 10.1210/clinem/dgad290 PMC10584003

[17] Eastwood SV, Mathur R, Atkinson M et al (2016) Algorithms for the capture and adjudication of prevalent and incident diabetes in UK biobank. PLOS ONE 11(9):e0162388. 10.1371/journal.pone.0162388 27631769 10.1371/journal.pone.0162388 PMC5025160

[18] Jørgensen T, Borch-Johnsen K, Thomsen TF, Ibsen H, Glümer C, Pisinger C (2003) A randomized non-pharmacological intervention study for prevention of ischaemic heart disease: baseline results Inter99 (1). Eur J Cardiovasc Prev Rehabil 10(5):377–386. 10.1097/01.hjr.0000096541.30533.82 14663300 10.1097/01.hjr.0000096541.30533.82

[19] Glümer C, Jørgensen T, Borch-Johnsen K (2003) Prevalences of diabetes and impaired glucose regulation in a Danish population. Diabetes Care 26(8):2335–2340. 10.2337/diacare.26.8.2335 12882858 10.2337/diacare.26.8.2335

[20] Nielsen JS, Thomsen RW, Steffensen C, Christiansen JS (2012) The Danish Centre for Strategic Research in Type 2 Diabetes (DD2) study: implementation of a nationwide patient enrollment system. Clin Epidemiol 4(Suppl 1):27–36. 10.2147/CLEP.S30838 23071409 10.2147/CLEP.S30838 PMC3469284

[21] Liu X, Jian X, Boerwinkle E (2011) dbNSFP: a lightweight database of human nonsynonymous SNPs and their functional predictions. Hum Mutat 32(8):894–899. 10.1002/humu.21517 21520341 10.1002/humu.21517 PMC3145015

[22] McLaren W, Gil L, Hunt SE et al (2016) The ensembl variant effect predictor. Genome Biol 17(1):122. 10.1186/s13059-016-0974-4 27268795 10.1186/s13059-016-0974-4 PMC4893825

[23] Flannick J, Mercader JM, Fuchsberger C et al (2019) Exome sequencing of 20,791 cases of type 2 diabetes and 24,440 controls. Nature 570(7759):71–76. 10.1038/s41586-019-1231-2 31118516 10.1038/s41586-019-1231-2 PMC6699738

[24] Sluiter WJ, Erkelens DW, Reitsma WD, Doorenbos H (1976) Glucose tolerance and insulin release, a mathematical approach I. Assay of the beta-cell response after oral glucose loading. Diabetes 25(4):241–244. 10.2337/diab.25.4.241 773721 10.2337/diab.25.4.241

[25] Hansen T, Drivsholm T, Urhammer SA et al (2007) The BIGTT test: a novel test for simultaneous measurement of pancreatic beta-cell function, insulin sensitivity, and glucose tolerance. Diabetes Care 30(2):257–262. 10.2337/dc06-1240 17259491 10.2337/dc06-1240

[26] Matthews DR, Hosker JP, Rudenski AS, Naylor BA, Treacher DF, Turner RC (1985) Homeostasis model assessment: insulin resistance and beta-cell function from fasting plasma glucose and insulin concentrations in man. Diabetologia 28(7):412–419. 10.1007/BF00280883 3899825 10.1007/BF00280883

[27] Stumvoll M, Van Haeften T, Fritsche A, Gerich J (2001) Oral glucose tolerance test indexes for insulin sensitivity and secretion based on various availabilities of sampling times. Diabetes Care 24(4):796–797. 10.2337/diacare.24.4.796 11315860 10.2337/diacare.24.4.796

[28] Lee S, Zhao Z, Miropolsky L, Wu M (2023) SKAT: SNP-Set (Sequence) Kernel Association Test. R package version 2.2.5. https://CRAN.R-project.org/package=SKAT

[29] Lee S, Emond MJ, Bamshad MJ et al (2012) Optimal unified approach for rare-variant association testing with application to small-sample case-control whole-exome sequencing studies. Am J Hum Genet 91(2):224–237. 10.1016/j.ajhg.2012.06.007 22863193 10.1016/j.ajhg.2012.06.007 PMC3415556

[30] R Core Team (2024) R: A Language and Environment for Statistical Computing. R Foundation for Statistical Computing, Vienna, Austria; R Foundation for Statistical Computing

[31] Viechtbauer W (2010) Conducting meta-analyses in R with the metafor package. J Stat Softw 36:1–48. 10.18637/jss.v036.i03

[32] Harder MN, Ribel-Madsen R, Justesen JM et al (2013) Type 2 diabetes risk alleles near BCAR1 and in ANK1 associate with decreased β-cell function whereas risk alleles near ANKRD55 and GRB14 associate with decreased insulin sensitivity in the Danish Inter99 cohort. J Clin Endocrinol Metab 98(4):E801–E806. 10.1210/jc.2012-4169 23457408 10.1210/jc.2012-4169

[33] Ørskov C, Jeppesen J, Madsbad S, Holst JJ (1991) Proglucagon products in plasma of noninsulin-dependent diabetics and nondiabetic controls in the fasting state and after oral glucose and intravenous arginine. J Clin Invest 87(2):415–423. 10.1172/JCI115012 1991827 10.1172/JCI115012 PMC295092

[34] Gao W, Liu L, Huh E et al (2023) Human GLP1R variants affecting GLP1R cell surface expression are associated with impaired glucose control and increased adiposity. Nat Metab 5(10):1673–1684. 10.1038/s42255-023-00889-6 37709961 10.1038/s42255-023-00889-6 PMC11610247

[35] Lotta LA, Mokrosiński J, Mendes de Oliveira E et al (2019) Human gain-of-function *MC4R* variants show signaling bias and protect against obesity. Cell 177(3):597-607.e9. 10.1016/j.cell.2019.03.044 31002796 10.1016/j.cell.2019.03.044 PMC6476272

[36] Kizilkaya HS, Sørensen KV, Madsen JS et al (2024) Characterization of genetic variants of *GIPR* reveals a contribution of β-arrestin to metabolic phenotypes. Nat Metab 6:1268–1281. 10.1038/s42255-024-01061-4 38871982 10.1038/s42255-024-01061-4 PMC11272584

[37] Fry A, Littlejohns TJ, Sudlow C et al (2017) Comparison of sociodemographic and health-related characteristics of UK biobank participants with those of the general population. Am J Epidemiol 186(9):1026–1034. 10.1093/aje/kwx246 28641372 10.1093/aje/kwx246 PMC5860371

[38] Karamitri A, Plouffe B, Bonnefond A et al (2018) Type 2 diabetes–associated variants of the MT2 melatonin receptor affect distinct modes of signaling. Sci Signal 11(545):eaan6622. 10.1126/scisignal.aan6622 30154102 10.1126/scisignal.aan6622

[39] Tuomi T, Nagorny CLF, Singh P et al (2016) Increased melatonin signaling is a risk factor for type 2 diabetes. Cell Metab 23(6):1067–1077. 10.1016/j.cmet.2016.04.009 27185156 10.1016/j.cmet.2016.04.009

[40] Costes S, Boss M, Thomas AP, Matveyenko AV (2015) Activation of melatonin signaling promotes β-cell survival and function. Mol Endocrinol 29(5):682–692. 10.1210/me.2014-1293 25695910 10.1210/me.2014-1293 PMC4415205

[41] Lee YH, Jung HS, Kwon MJ et al (2020) Melatonin protects INS-1 pancreatic β-cells from apoptosis and senescence induced by glucotoxicity and glucolipotoxicity. Islets 12(4):87–98. 10.1080/19382014.2020.1783162 32673151 10.1080/19382014.2020.1783162 PMC7527021

[42] Lauritzen ES, Kampmann U, Pedersen MGB et al (2022) Three months of melatonin treatment reduces insulin sensitivity in patients with type 2 diabetes-A randomized placebo-controlled crossover trial. J Pineal Res 73(1):e12809. 10.1111/jpi.12809 35619221 10.1111/jpi.12809 PMC9540532

[43] Kampmann U, Lauritzen ES, Grarup N et al (2021) Acute metabolic effects of melatonin—A randomized crossover study in healthy young men. J Pineal Res 70(2):e12706. 10.1111/jpi.12706 33220095 10.1111/jpi.12706

[44] Ramracheya RD, Muller DS, Squires PE et al (2008) Function and expression of melatonin receptors on human pancreatic islets. J Pineal Res 44(3):273–279. 10.1111/j.1600-079X.2007.00523.x 18194202 10.1111/j.1600-079X.2007.00523.x

[45] Lauritzen ES, Støy J, Bæch-Laursen C et al (2021) The effect of melatonin on incretin hormones: results from experimental and randomized clinical studies. J Clin Endocrinol Metab 106(12):e5109–e5123. 10.1210/clinem/dgab521 34265066 10.1210/clinem/dgab521

[46] Kedziora-Kornatowska K, Szewczyk-Golec K, Kozakiewicz M et al (2009) Melatonin improves oxidative stress parameters measured in the blood of elderly type 2 diabetic patients. J Pineal Res 46(3):333–337. 10.1111/j.1600-079X.2009.00666.x 19317795 10.1111/j.1600-079X.2009.00666.x

[47] Raygan F, Ostadmohammadi V, Bahmani F, Reiter RJ, Asemi Z (2019) Melatonin administration lowers biomarkers of oxidative stress and cardio-metabolic risk in type 2 diabetic patients with coronary heart disease: a randomized, double-blind, placebo-controlled trial. Clin Nutr 38(1):191–196. 10.1016/j.clnu.2017.12.004 29275919 10.1016/j.clnu.2017.12.004

[48] Możdżan M, Możdżan M, Chałubiński M, Wojdan K, Broncel M (2014) The effect of melatonin on circadian blood pressure in patients with type 2 diabetes and essential hypertension. Arch Med Sci 10(4):669–675. 10.5114/aoms.2014.44858 25276149 10.5114/aoms.2014.44858 PMC4175768

[49] Delpino FM, Figueiredo LM, Nunes BP (2021) Effects of melatonin supplementation on diabetes: a systematic review and meta-analysis of randomized clinical trials. Clin Nutr 40(7):4595–4605. 10.1016/j.clnu.2021.06.007 34229264 10.1016/j.clnu.2021.06.007

