# Supplementary material for: Rare MTNR1B variants causing diminished MT2 signalling associate with elevated HbA1c levels but not with type 2 diabetes
Source: Diabetologia. 2025 Mar 10;68(5):1016–30. doi: 10.1007/s00125-025-06381-y (PMC12021717; doi:10.1007/s00125-025-06381-y)
Supplement: Supplementary file 2 — Supplementary file2 (PDF 1024 KB) [file 125_2025_6381_MOESM2_ESM.pdf]

ESM Table 5 | Group-based burden testing of *MTNR1B* variant groups in the UK Biobank

Phenotype	Non-carriers	Carriers	Estimate	Standard error	P regression model	P SKAT-O	P SKAT	Variant group
HbA_{1c}	283,250	1585	0.038544634	0.024145008	0.110404837	0.178517491	0.101711734	Synonymous
Random glucose	247,410	1401	-0.021891419	0.026500348	0.408759252	0.613927849	0.391781023	Synonymous
Type 2 diabetes	292,452	1608	-0.278818028	0.127091472	0.028247099	0.040349426	0.022755698	Synonymous
Fat percentage	322,712	1785	0.031552931	0.017806142	0.076391326	0.188900222	0.107846394	Synonymous
Diastolic BP	299,940	1652	0.033939114	0.024308996	0.162669014	0.3664816	0.230147198	Synonymous
Systolic BP	299,933	1652	0.007232638	0.023037044	0.753554009	0.717241091	0.70783111	Synonymous
Triglycerides	313,356	1739	-0.022879567	0.023389259	0.32797205	0.186571423	0.429710721	Synonymous
Cholesterol	313,606	1739	0.048504572	0.023675882	0.040493143	0.012921147	0.018896329	Synonymous
LDL	313,010	1737	0.044438381	0.023994698	0.064025553	0.021568303	0.03091608	Synonymous
HDL	287,059	1599	0.040753322	0.022705433	0.072675308	0.209837369	0.120108281	Synonymous
Hip circ.	328,061	1812	0.027944085	0.023529495	0.234984239	0.405986312	0.241641579	Synonymous
Waist circ.	328,094	1812	0.027270695	0.020659294	0.186829041	0.351628571	0.206672741	Synonymous
Weight	327,709	1813	0.034209222	0.020655841	0.097691892	0.206933938	0.118466835	Synonymous
BMI	327,591	1812	0.017945099	0.023355159	0.442275698	0.700185864	0.469253055	Synonymous
HbA_{1c}	261,848	22,987	-0.017996617	0.006639148	0.006714905	0.010816968	0.007951152	WT-like excl. G24E
Random glucose	228,770	20,041	0.006452897	0.007341511	0.379423682	0.475085766	0.35598381	WT-like excl. G24E
Type 2 diabetes	270,393	23,667	-0.064002499	0.031914628	0.044917277	0.020244746	0.081291378	WT-like excl. G24E
Fat percentage	298,306	26,191	-0.002041714	0.004868116	0.674920103	0.049056537	0.5188228	WT-like excl. G24E
Diastolic BP	277,141	24,451	0.00577849	0.006616389	0.382467546	0.796854393	0.668079423	WT-like excl. G24E
Systolic BP	277,135	24,450	0.000820614	0.006270318	0.89587609	1	0.928843938	WT-like excl. G24E
Triglycerides	289,684	25,411	-0.006501688	0.006407457	0.310246588	0.41757373	0.300015712	WT-like excl. G24E
Cholesterol	289,916	25,429	-0.004390844	0.006483711	0.498272192	0.81141044	0.687059909	WT-like excl. G24E
LDL	289,360	25,387	-0.004350291	0.006572781	0.508058458	1	0.820929152	WT-like excl. G24E
HDL	265,386	23,272	-0.004253679	0.006236646	0.495210442	0.335315351	0.442947591	WT-like excl. G24E
Hip circ.	303,224	26,649	-0.004558133	0.00642602	0.478123636	0.073205724	0.364590304	WT-like excl. G24E
Waist circ.	303,256	26,650	-0.003418935	0.005642045	0.544532111	0.016096975	0.4931768	WT-like excl. G24E

Weight	302,911	26,611	-0.003660997	0.005646658	0.516760669	0.010964852	0.329064661	WT-like excl. G24E
BMI	302,805	26,598	0.000685282	0.006384349	0.914521104	0.080728186	0.94877831	WT-like excl. G24E
HbA_{1c}	238,248	46,558	-0.009273854	0.004538462	0.041014624	NA	NA	G24E
Random glucose	208,070	40,713	0.005380513	0.0050159	0.283410638	NA	NA	G24E
Type 2 diabetes	245,911	48,119	0.008402775	0.021213091	0.692021888	NA	NA	G24E
Fat percentage	271,334	53,131	0.003387391	0.003324001	0.308170289	NA	NA	G24E
Diastolic BP	252,062	49,501	0.007566594	0.004523991	0.094417285	NA	NA	G24E
Systolic BP	252,055	49,501	0.002521329	0.004287256	0.556466596	NA	NA	G24E
Triglycerides	263,526	51,537	-0.009740123	0.004373085	0.025928689	NA	NA	G24E
Cholesterol	263,715	51,598	-0.007015287	0.004424272	0.112822843	NA	NA	G24E
LDL	263,238	51,477	-0.006339604	0.004486111	0.157608992	NA	NA	G24E
HDL	241,245	47,382	-0.001377908	0.004252541	0.745923244	NA	NA	G24E
Hip circ.	275,823	54,018	0.003932786	0.004389169	0.370242405	NA	NA	G24E
Waist circ.	275,853	54,021	0.005918037	0.003853509	0.124599999	NA	NA	G24E
Weight	275,531	53,959	0.002505905	0.00385606	0.51578201	NA	NA	G24E
BMI	275,432	53,939	0.006070224	0.004359686	0.163816301	NA	NA	G24E
HbA_{1c}	282,392	2443	0.058000337	0.019464844	0.002885096	0.002940991	0.002563357	Missense excl. WT-like
Random glucose	246,704	2107	0.021092944	0.021647717	0.329872472	0.481453592	0.301508498	Missense excl. WT-like
Type 2 diabetes	291,530	2530	0.0419579	0.089580281	0.639510571	0.85996948	0.68091622	Missense excl. WT-like
Fat percentage	321,698	2799	0.015092889	0.01422712	0.288757328	0.374740417	0.272725014	Missense excl. WT-like
Diastolic BP	298,991	2601	-0.007641363	0.019385647	0.693451151	0.529507625	0.722000446	Missense excl. WT-like
Systolic BP	298,985	2600	0.009882887	0.018374749	0.590679967	0.203828323	0.604799361	Missense excl. WT-like
Triglycerides	312,381	2714	0.006505271	0.018734989	0.728421956	0.729534379	0.676297223	Missense excl. WT-like
Cholesterol	312,625	2720	-0.003407506	0.018944031	0.857252973	1	0.829348268	Missense excl. WT-like
LDL	312,031	2716	-0.008840272	0.019202189	0.645244993	0.803804204	0.602622326	Missense excl. WT-like
HDL	286,190	2468	0.005929842	0.018303509	0.745958754	0.771158391	0.706879838	Missense excl. WT-like
Hip circ.	327,024	2849	0.025699111	0.018778419	0.171142499	0.057587373	0.177795241	Missense excl. WT-like
Waist circ.	327,058	2848	0.012430238	0.016490676	0.450985671	0.320905918	0.429821516	Missense excl. WT-like
Weight	326,678	2844	0.019579735	0.016504011	0.23548015	0.151294606	0.219854686	Missense excl. WT-like
BMI	326,560	2843	0.038452392	0.018658729	0.039320434	0.011035286	0.044378766	Missense excl. WT-like

HbA_{1c}	283,301	1534	0.08685642	0.024502099	0.00039289	0.000709961	0.000557915	Impaired-function
Random glucose	247,490	1321	0.018051654	0.027267501	0.507958488	0.661479425	0.488161447	Impaired-function
Type 2 diabetes	292,474	1586	-0.022096405	0.115593361	0.848403099	1	0.832868177	Impaired-function
Fat percentage	322,754	1743	0.022198342	0.017983158	0.217056189	0.322855258	0.211652347	Impaired-function
Diastolic BP	299,974	1618	0.012506921	0.02451768	0.60996885	0.587486946	0.599416964	Impaired-function
Systolic BP	299,968	1617	0.031972151	0.023241806	0.168936904	0.252226616	0.17151399	Impaired-function
Triglycerides	313,403	1692	0.014632544	0.023669836	0.536448096	0.674711721	0.501677028	Impaired-function
Cholesterol	313,650	1695	0.003296233	0.02393911	0.890483624	1	0.884632881	Impaired-function
LDL	313,054	1693	-0.007356134	0.024261778	0.761738799	1	0.769119527	Impaired-function
HDL	287,111	1547	0.032093278	0.023060687	0.164018027	0.247071771	0.160154543	Impaired-function
Hip circ.	328,092	1781	0.055358442	0.023693229	0.019467524	0.025894047	0.017375456	Impaired-function
Waist circ.	328,127	1779	0.026353818	0.020814782	0.205474358	0.277934625	0.180601359	Impaired-function
Weight	327,746	1776	0.036601488	0.020834476	0.078957085	0.097661404	0.063767747	Impaired-function
BMI	327,628	1775	0.0619932	0.023557078	0.008498363	0.007169354	0.00725616	Impaired-function
HbA_{1c}	283,545	1290	0.052589498	0.026709767	0.048962803	0.026806542	0.041001111	pLoF excl. WT-like
Random glucose	247,697	1114	0.033913942	0.029682675	0.253226557	0.336398715	0.226851833	pLoF excl. WT-like
Type 2 diabetes	292,718	1342	0.077901292	0.120517057	0.518025359	0.700037407	0.537614821	pLoF excl. WT-like
Fat percentage	323,018	1479	0.018436547	0.019521032	0.344942837	0.324438326	0.339350965	pLoF excl. WT-like
Diastolic BP	300,220	1372	-0.011747634	0.026615552	0.65893657	0.53614792	0.572519266	pLoF excl. WT-like
Systolic BP	300,214	1371	0.023255325	0.025231952	0.356705768	0.254838438	0.384176868	pLoF excl. WT-like
Triglycerides	313,655	1440	0.00966071	0.025648807	0.706431471	0.695396778	0.695389775	pLoF excl. WT-like
Cholesterol	313,904	1441	0.004480841	0.025954506	0.862932851	1	0.917548862	pLoF excl. WT-like
LDL	313,308	1439	0.004561186	0.026307065	0.862350839	1	0.924421659	pLoF excl. WT-like
HDL	287,344	1314	0.006944607	0.025013249	0.781291114	0.749954174	0.754190019	pLoF excl. WT-like
Hip circ.	328,366	1507	0.029187548	0.025748209	0.256973332	0.05120849	0.253560149	pLoF excl. WT-like
Waist circ.	328,401	1505	0.017300375	0.022622329	0.444422333	0.272917703	0.424186002	pLoF excl. WT-like
Weight	328,018	1504	0.025744602	0.022632297	0.25532343	0.150461816	0.25167009	pLoF excl. WT-like
BMI	327,900	1503	0.050659241	0.02559111	0.04775337	0.012741288	0.050711084	pLoF excl. WT-like
HbA_{1c}	282,980	1855	0.071451511	0.022298977	0.001354295	0.002042063	0.001418438	pLoF & impaired-function
Random glucose	247,213	1598	0.03142483	0.024811677	0.205323183	0.28629711	0.177361919	pLoF & impaired-function

Type 2 diabetes	292,136	1924	0.035382577	0.102333348	0.729524569	0.886811109	0.746886877	pLoF & impaired-function
Fat percentage	322,375	2122	0.017764117	0.016311689	0.276136092	0.414917753	0.266371938	pLoF & impaired-function
Diastolic BP	299,624	1968	-0.002365275	0.022248231	0.915334114	0.555548768	0.872789057	pLoF & impaired-function
Systolic BP	299,618	1967	0.027792764	0.021089297	0.187551147	0.253661048	0.205114497	pLoF & impaired-function
Triglycerides	313,034	2061	0.011984051	0.021463126	0.576602154	0.725950295	0.535042666	pLoF & impaired-function
Cholesterol	313,281	2064	0.001127176	0.021710719	0.958594116	1	0.998311564	pLoF & impaired-function
LDL	312,686	2061	-0.006713593	0.022006349	0.76030903	0.867509231	0.717123779	pLoF & impaired-function
HDL	286,775	1883	0.02445366	0.020918729	0.242411039	0.368667153	0.232974602	pLoF & impaired-function
Hip circ.	327,707	2166	0.033459246	0.021501138	0.119670624	0.057986838	0.109334146	pLoF & impaired-function
Waist circ.	327,742	2164	0.018724122	0.018887033	0.32150348	0.375355853	0.277640005	pLoF & impaired-function
Weight	327,361	2161	0.029136764	0.01890212	0.123207102	0.139202513	0.10237761	pLoF & impaired-function
BMI	327,243	2160	0.048402987	0.021371169	0.023521111	0.010908216	0.022096934	pLoF & impaired-function

ESM Table 5 | Estimate refers to the beta as SD of the phenotype. p-values (P) are shown for the regression model (linear for quantitative traits, and logistic for case-control study), SKAT-O, and SKAT in separate columns. HbA_{1c}, glycated hemoglobin A_{1c}; circ., circumference; BP, blood pressure; LDL, low-density lipoprotein; HDL, high-density lipoprotein; BMI, body mass index. WT, wild-type; pLoF, predicted loss of function; Impaired-function, impaired-function variants.

ESM Table 6 | Group-based burden testing of *MTNR1B* variant groups in the Danish population

Phenotype	Non-carriers	Carriers	Estimate	Standard error	P regression model	P SKAT-O	P SKAT	Variant group
CIR	5301	37	-0.114419513	0.154983663	0.460383978	0.620426275	0.434482447	Synonymous
Stumvoll-ISI	5283	36	-0.029106426	0.166252434	0.861028408	1	0.877457649	Synonymous
BIGTT-AIR	5051	34	-0.161431701	0.16778866	0.336038516	0.482017635	0.316386709	Synonymous
BIGTT-SI	5051	34	-0.105637979	0.163981847	0.519471403	0.721753812	0.529571087	Synonymous
HOMA-IR	5460	38	-0.119730546	0.161849632	0.459474781	0.646831609	0.455779221	Synonymous
HbA_{1c}	5665	40	0.133193108	0.148210128	0.368862445	0.527188746	0.35464895	Synonymous
Insulin 120 min	5479	38	0.08623649	0.16067752	0.591492812	0.801892472	0.627224931	Synonymous
Insulin 30 min	5368	37	-0.117625174	0.164556075	0.474761707	0.667326128	0.478627903	Synonymous
Fasting insulin	5463	38	-0.161305466	0.162602107	0.321228693	0.478770394	0.314825683	Synonymous
Glucose 120 min	5649	40	0.094618852	0.156282718	0.54491493	0.749836107	0.565058357	Synonymous
Glucose 30 min	5557	40	-0.009542644	0.14550701	0.947712948	0.824354067	0.982725898	Synonymous
Fasting glucose	5666	40	0.192056107	0.143831934	0.181837603	0.248031912	0.155349741	Synonymous
Type 2 diabetes	7055	53	-0.045331516	0.411901063	0.912366248	0.751328602	0.558765194	Synonymous
CIR	4886	452	0.029500492	0.046481844	0.525672875	0.201247602	0.411964736	WT-like excl. G24E
Stumvoll-ISI	4877	442	0.039919676	0.049610132	0.421047426	0.51640909	0.376651373	WT-like excl. G24E
BIGTT-AIR	4667	418	0.035351014	0.050036594	0.47990769	0.215905267	0.392571747	WT-like excl. G24E
BIGTT-SI	4667	418	0.038340455	0.048896542	0.4330099	0.492466337	0.356677708	WT-like excl. G24E
HOMA-IR	5031	467	-0.008942654	0.048400173	0.853420257	0.859444844	0.753770664	WT-like excl. G24E
HbA_{1c}	5221	484	-0.070365367	0.044647388	0.115075745	0.022704607	0.113492425	WT-like excl. G24E
Insulin 120 min	5061	456	-0.065078416	0.048473071	0.179467081	0.226411528	0.157572331	WT-like excl. G24E
Insulin 30 min	4948	457	-0.011559571	0.049080005	0.813810166	0.763533074	0.834868598	WT-like excl. G24E
Fasting insulin	5033	468	0.004362766	0.048579291	0.928443769	1	0.98599968	WT-like excl. G24E
Glucose 120 min	5206	483	-0.083578121	0.047121405	0.076170269	0.080787873	0.058519319	WT-like excl. G24E
Glucose 30 min	5126	471	-0.008109633	0.044426328	0.85516469	0.481539499	0.6548596	WT-like excl. G24E
Fasting glucose	5222	484	-0.100299537	0.043313679	0.020612452	0.005870428	0.006146813	WT-like excl. G24E
Type 2 diabetes	6485	623	-0.031929083	0.114624259	0.780586872	1	0.788994653	WT-like excl. G24E
CIR	4369	967	0.030027621	0.031233914	0.336405396	NA	NA	G24E

Stumvoll-ISI	4355	960	-0.028556634	0.033099455	0.388312612	NA	NA	G24E
BIGTT-AIR	4165	918	0.097811669	0.033180982	0.003214782	NA	NA	G24E
BIGTT-SI	4165	918	-0.044991434	0.032447511	0.165627352	NA	NA	G24E
HOMA-IR	4492	1002	-0.003888933	0.032475692	0.904686332	NA	NA	G24E
HbA_{1c}	4668	1033	-0.035671198	0.030083827	0.235779722	NA	NA	G24E
Insulin 120 min	4525	988	0.031698634	0.032429673	0.328385558	NA	NA	G24E
Insulin 30 min	4425	978	0.066927205	0.03298038	0.042476366	NA	NA	G24E
Fasting insulin	4495	1002	0.008947656	0.032625545	0.783900459	NA	NA	G24E
Glucose 120 min	4656	1029	0.035343178	0.031777542	0.266096616	NA	NA	G24E
Glucose 30 min	4575	1020	0.011137263	0.029720762	0.707875448	NA	NA	G24E
Fasting glucose	4669	1033	-0.057163144	0.029189199	0.0502358	NA	NA	G24E
Type 2 diabetes	5895	1209	-0.164372897	0.082571253	0.046516139	NA	NA	G24E
CIR	5295	43	-0.07456308	0.143640479	0.603715924	0.045089173	0.614464556	Missense excl. WT-like
Stumvoll-ISI	5272	47	0.27428794	0.145431797	0.059346164	0.10179657	0.059350061	Missense excl. WT-like
BIGTT-AIR	5043	42	-0.221890527	0.150878783	0.14144661	0.045974213	0.145006923	Missense excl. WT-like
BIGTT-SI	5043	42	0.247472194	0.147438711	0.093315944	0.158382678	0.094474071	Missense excl. WT-like
HOMA-IR	5450	48	-0.193168485	0.143943818	0.179660771	0.262238022	0.180117556	Missense excl. WT-like
HbA_{1c}	5657	48	0.318038052	0.135178176	0.018669872	0.033855618	0.018831448	Missense excl. WT-like
Insulin 120 min	5470	47	-0.172220882	0.144417302	0.233107795	0.366406735	0.233133014	Missense excl. WT-like
Insulin 30 min	5362	43	-0.101113559	0.152516121	0.507378306	0.1287516	0.505721837	Missense excl. WT-like
Fasting insulin	5453	48	-0.192912179	0.144619001	0.182280843	0.192547076	0.18308095	Missense excl. WT-like
Glucose 120 min	5641	48	-0.270976088	0.142558598	0.05737813	0.099723763	0.057854502	Missense excl. WT-like
Glucose 30 min	5550	47	-0.021000616	0.134157904	0.875615762	1	0.874519937	Missense excl. WT-like
Fasting glucose	5658	48	-0.085398766	0.131255318	0.515311011	0.706168474	0.507847658	Missense excl. WT-like
Type 2 diabetes	7037	71	0.413901513	0.326140359	0.204408917	0.355740256	0.220029859	Missense excl. WT-like
CIR	5309	29	-0.410232725	0.174638232	0.018858045	0.032771063	0.019744826	Impaired-function
Stumvoll-ISI	5285	34	0.293425153	0.17080109	0.085866779	0.135924935	0.086430969	Impaired-function
BIGTT-AIR	5056	29	-0.465011088	0.181300193	0.01034999	0.017577303	0.010980963	Impaired-function
BIGTT-SI	5056	29	0.288770324	0.177246345	0.103332036	0.16307052	0.105240654	Impaired-function
HOMA-IR	5464	34	-0.357663125	0.17079257	0.036293475	0.059625076	0.036406787	Impaired-function

HbA_{1c}	5671	34	0.360261269	0.160443651	0.024780219	0.04146729	0.024954236	Impaired-function
Insulin 120 min	5483	34	-0.148342582	0.169617326	0.381844895	0.502559202	0.38463386	Impaired-function
Insulin 30 min	5376	29	-0.393440083	0.185454605	0.033926042	0.055422573	0.033715256	Impaired-function
Fasting insulin	5467	34	-0.38997756	0.171581413	0.02307412	0.038673725	0.023200973	Impaired-function
Glucose 120 min	5655	34	-0.136580494	0.169240046	0.419687408	0.567475807	0.421511807	Impaired-function
Glucose 30 min	5564	33	0.100651882	0.15992618	0.529136091	0.694091281	0.528772403	Impaired-function
Fasting glucose	5672	34	0.045064046	0.155785417	0.772385299	1	0.778588789	Impaired-function
Type 2 diabetes	7062	46	0.50639916	0.391124972	0.195415217	0.29667047	0.193112515	Impaired-function
CIR	5320	18	0.465148155	0.221422983	0.035712221	0.056913714	0.038347973	pLoF excl. WT-like
Stumvoll-ISI	5298	21	-0.050073862	0.21713697	0.817627019	0.857290052	0.837009194	pLoF excl. WT-like
BIGTT-AIR	5067	18	0.323052412	0.229934268	0.160089229	0.253819986	0.171131312	pLoF excl. WT-like
BIGTT-SI	5067	18	0.026929781	0.224749549	0.904629457	0.828080548	0.89232438	pLoF excl. WT-like
HOMA-IR	5477	21	0.058107272	0.217161561	0.78903558	0.719345511	0.795222549	pLoF excl. WT-like
HbA_{1c}	5684	21	0.342598336	0.203969251	0.093078944	0.130479365	0.089205605	pLoF excl. WT-like
Insulin 120 min	5496	21	0.158215894	0.215586259	0.463049009	0.632495612	0.485440077	pLoF excl. WT-like
Insulin 30 min	5387	18	0.262211263	0.235185289	0.264937117	0.378575325	0.262945465	pLoF excl. WT-like
Fasting insulin	5480	21	0.087399249	0.218178342	0.688740334	0.708632064	0.700718284	pLoF excl. WT-like
Glucose 120 min	5668	21	-0.011285265	0.215120692	0.958163886	0.680167288	0.940175843	pLoF excl. WT-like
Glucose 30 min	5577	20	-0.239229956	0.205164515	0.243648176	0.371352993	0.259307341	pLoF excl. WT-like
Fasting glucose	5685	21	-0.160535781	0.197998896	0.417519398	0.59756946	0.451624668	pLoF excl. WT-like
Type 2 diabetes	7076	32	0.354767064	0.510348441	0.486963335	0.710388014	0.568828057	pLoF excl. WT-like
CIR	5303	35	-0.126928467	0.15912244	0.425092918	0.032878614	0.424876443	pLoF & impaired-function
Stumvoll-ISI	5279	40	0.229703578	0.157577658	0.144977782	0.222727318	0.14178898	pLoF & impaired-function
BIGTT-AIR	5050	35	-0.249643751	0.165189419	0.130785089	0.034667212	0.130507967	pLoF & impaired-function
BIGTT-SI	5050	35	0.21535527	0.161441478	0.182279243	0.282686068	0.18222412	pLoF & impaired-function
HOMA-IR	5458	40	-0.224958624	0.157589178	0.15349257	0.213408491	0.152425602	pLoF & impaired-function
HbA_{1c}	5665	40	0.33330521	0.148005004	0.024361077	0.041022942	0.02379169	pLoF & impaired-function
Insulin 120 min	5477	40	-0.099701021	0.1564752	0.524041769	0.593429958	0.513202365	pLoF & impaired-function
Insulin 30 min	5370	35	-0.16131794	0.168954668	0.339721541	0.09602541	0.339692563	pLoF & impaired-function
Fasting insulin	5461	40	-0.23983688	0.158324368	0.129869194	0.150814017	0.127927926	pLoF & impaired-function

Glucose 120 min	5649	40	-0.179368357	0.156110744	0.25061198	0.374009614	0.246345625	pLoF & impaired-function
Glucose 30 min	5558	39	-0.004927525	0.147197108	0.973296445	1	0.988384828	pLoF & impaired-function
Fasting glucose	5666	40	-0.008336431	0.14370944	0.953743546	1	0.975047918	pLoF & impaired-function
Type 2 diabetes	7052	56	0.536158573	0.362810674	0.139463562	0.238518243	0.147503268	pLoF & impaired-function

ESM Table 6 | Estimate refers to the beta as SD of the phenotype. p-values (P) are shown for the regression model (linear for quantitative traits, and logistic for case-control study), SKAT-O, and SKAT in separate columns. CIR, corrected insulin response; Stumvoll-ISI, Stumvoll insulin sensitivity index; BIGTT-AIR, the beta cell function insulin sensitivity glucose tolerance test (BIGTT) – acute insulin response; BIGTT-SI, the BIGTT – insulin sensitivity index; HOMA-IR, the homeostasis model assessment of insulin resistance index; HbA_{1c}, glycated hemoglobin A_{1c}. Time points for some of the phenotypes refer to the times during an OGTT. WT, wild-type; pLoF, predicted loss of function; Impaired-function, impaired-function variants.

ESM Table 7 | Group-based burden testing for type 2 diabetes prevalence in the Danish population

**Variant group**	**Type 2 diabetes cases**		**Controls**		**OR (95% CI)**	**p-value**
	Carriers	Non-carriers	Carriers	Non-carriers		
Synonymous	19	2911	34	4209	0.96 (0.43, 2.14)	0.75
WT-like excl. G24E	251	2679	376	3867	0.97 (0.77, 1.21)	0.79
Missense excl. WT-like	31	2899	40	4203	1.51 (0.80, 2.87)	0.36
Impaired-function	20	2910	26	4217	1.66 (0.77, 3.57)	0.19
pLoF excl. WT-like	15	2915	17	4226	1.42 (0.52, 3.88)	0.71

ESM Table 7 | The count of carriers and non-carriers represents the numbers prior to analysis, hence the number in the burden test may be slightly lower as we exclude individuals having missing values in any of the co-variates. WT, wild-type; impaired-function, impaired-function variants; pLoF, predicted loss of function; OR, odds ratio; CI, confidence interval. p-value derived from burden testing (SKAT or SKAT-O).

ESM Table 8 | Characteristics of the participants at baseline (first visit)

Phenotypes		All (N = 36)	rs10830963 carriers (N = 13)	Impaired-function variant carriers (N = 7)	Non-carriers (N = 16)
		Mean ± *SE*	*Mean* ± *SE*	*Mean* ± *SE*	*Mean* ± *SE*
Age (years)		64.2 ± 1.3	64.4 ± 2.1	68.8 ± 1.9	62.1 ± 2.0
BMI (kg/m2)		25.3 ± 0.80	26.2 ± 1.1	24.3 ± 1.8	25.0 ± 1.4
Fat %		28.5 ± 1.7	29.7 ± 2.6	25.2 ± 3.3	28.8 ± 2.8
Waist circ. (cm)		95.3 ± 2.4	96.3 ± 3.6	95.2 ± 4.0	94.6 ± 4.3
Hip circ. (cm)		102.7 ± 1.6	104.8 ± 2.3	98.6 ± 2.6	102.7 ± 2.9
Systolic BP (mmHg)		129.3 ± 2.3	136.7 ± 2.7	130.1 ± 6.3	123.0 ± 3.2
Diastolic BP (mmHg)		75.8 ± 1.2	78.5 ± 1.3	76.1 ± 3.1	73.6 ± 2.0
Pulse (bpm)		63.7 ± 1.8	67.8 ± 3.9	61.9 ± 2.4	61.2 ± 2.0
HbA_{1c}	(%)	5.6 ± 0.091	5.6 ± 0.063	5.7 ± 0.11	5.4 ± 0.19
	(mmol/mol)	37.2 ± 0.99	38.0 ± 0.69	38.7 ± 1.2	35.9 ± 2.1
Total cholesterol (mmol/l)		5.5 ± 0.12	5.5 ± 0.17	5.5 ± 0.22	5.4 ± 0.22
LDL (mmol/l)		3.3 ± 0.10	3.3 ± 0.16	3.4 ± 0.25	3.3 ± 0.17
HDL (mmol/l)		1.6 ± 0.10	1.5 ± 0.15	1.5 ± 0.10	1.6 ± 0.20
Triglycerides (mmol/l)		1.3 ± 0.10	1.5 ± 0.18	1.2 ± 0.076	1.3 ± 0.16

ESM Table 8 | Impaired-function variant carriers refer to carriers of the *MTNR1B* variants impairing MT2 signaling. Non-carriers refer to controls not carrying any of the examined *MTNR1B* variants. Abbreviations: BMI, body mass index; circ, circumference; BP, blood pressure; HbA_{1c}, glycated hemoglobin A_{1c}; LDL, lower-density lipoprotein; HDL, high-density lipoprotein; TSH, thyroid-stimulating hormone, thyrotropin; ALAT, alanine aminotransferase; SE, standard error.

ESM Table 9 | The effect of melatonin on the glucose regulation

Phenotype	Estimate (95% CI)	p-value	Test
$AUC_{glucose}$ (mmol/l x min)	66.9 (25.4, 108.3)	0.0024	paired t-test
$iAUC_{glucose}$ (mmol/l x min)	33.1 (12.2, 54.1)	0.0029	paired t-test
$AUC_{insulin}$ (pmol/l x min)	-10390.5 (-17895.3, -2885.7)	0.00044	paired wilcoxon test *
$iAUC_{insulin}$ (pmol/l x min)	-4183.2 (-7706.1, -660.3)	0.0096	paired wilcoxon test *
AUC_{GIP} (pmol/l x min)	-1530.1 (-2380.6, -679.5)	0.00086	paired t-test
$iAUC_{GIP}$ (pmol/l x min)	-780.1 (-1256.2, -304.1)	0.0021	paired t-test
AUC_{GLP-1} (pmol/l x min)	58.7 (-172.3, 289.8)	0.41	paired wilcoxon test *
$iAUC_{GLP-1}$ (pmol/l x min)	85.1 (-48.6, 218.7)	0.20	paired t-test
$AUC_{glucagon}$ (pmol/l x min)	-111.3 (-214.5, -8.1)	0.035	paired t-test
$iAUC_{glucagon}$ (pmol/l x min)	3.0 (-14.9, 20.9)	0.74	paired t-test

ESM Table 9 | Abbreviations: iAUC, incremental AUC; GIP, glucose-dependent insulinotropic polypeptide; GLP-1, glucagon-like peptide-1. * Estimate and 95% CI derived from t-test since non-parametric tests do not provide that.

ESM Table 10 | Genetically determined differences in the glucose regulation at visit 2

Phenotype	Impaired-function vs Non		rs10830963 vs Non		Test
	**Estimate (95% CI)**	**p-value**	**Estimate (95% CI)**	**p-value**	
$AUC_{glucose}$ (mmol/l x min)	137.7 (-83.5, 358.9)	0.21	161.3 (19.2, 303.5)	0.028	Unpaired t-test
$iAUC_{glucose}$ (mmol/l x min)	68.4 (-15.4, 152.2)	0.10	58.1 (7.1, 109.1)	0.027	Unpaired t-test
$AUC_{insulin}$ (pmol/l x min)	-6840.0 (-43391.4, 29711.4)	0.99	4600.2 (-23879.0, 33079.5)	0.48	Unpaired Wilcoxon test *
$iAUC_{insulin}$ (pmol/l x min)	-2826.1 (-18079.4, 12427.2)	0.86	2451.6 (-9770.8, 14674.0)	0.53	Unpaired Wilcoxon test *
AUC_{GIP} (pmol/l x min)	-891.0 (-5088.8, 3306.8)	0.66	-808.5 (-3853.2, 2236.2)	0.59	Unpaired t-test
$iAUC_{GIP}$ (pmol/l x min)	-349.6 (-2398.5, 1699.3)	0.73	-428.6 (-1898.0, 1040.8)	0.56	Unpaired t-test
AUC_{GLP-1} (pmol/l x min)	164.7 (-588.3, 917.7)	0.99	-84.7 (-654.7, 485.2)	0.37	Unpaired Wilcoxon test *
$iAUC_{GLP-1}$ (pmol/l x min)	110.7 (-261.8, 483.2)	0.54	-27.7 (-329.0, 273.7)	0.36	Unpaired Wilcoxon test *
$AUC_{glucagon}$ (pmol/l x min)	-43.2 (-415.8, 329.4)	0.85	146.6 (-165.1, 458.2)	0.25	Unpaired Wilcoxon test *
$iAUC_{glucagon}$ (pmol/l x min)	-23.5 (-53.4, 6.4)	0.074	8.0 (-20.0, 36.0)	0.96	Unpaired Wilcoxon test *
CIR	-476.0 (-927.6, -24.4)	0.033	-390.8 (-740.1, -41.6)	0.028	Unpaired Wilcoxon test *
BIGTT-AIR	-528.1 (-1615.3, 559.1)	0.49	-479.4 (-1294.4, 335.5)	0.11	Unpaired Wilcoxon test *
$AUC30_{insulin}$ (pmol/l x min)	-1169.9 (-4509.4, 2169.5)	0.45	-288.1 (-3079.2, 2503.1)	0.96	Unpaired Wilcoxon test *
BIGTT-SI	-1.7 (-6.9, 3.5)	0.50	-2.5 (-5.8, 0.89)	0.14	Unpaired Welch's t-test
HOMA-IR	-0.29 (-3.5, 3.0)	0.59	-0.099 (-2.3, 2.1)	0.37	Unpaired Wilcoxon test *

ESM Table 10 | "Impaired-function" refers to impaired-function variant carriers. "rs10830963" refers to homozygous carriers of rs10830963. "Non" refers to non-carriers. Abbreviations: iAUC, incremental AUC; GIP, glucose-dependent insulinotropic polypeptide; GLP-1, glucagon-like peptide-1; CIR, corrected insulin response; BIGTT-AIR, the beta cell function insulin sensitivity glucose tolerance test (BIGTT) – acute insulin response; AUC30, AUC at time 0–30 of an oral glucose tolerance test; BIGTT-SI, BIGTT – insulin sensitivity index; HOMA-IR, homeostatic model assessment for insulin resistance. * Estimate and 95% CI derived from t-test since non-parametric tests do not provide that.

ESM Table 11 | Difference-in-differences of phenotypes related to glucose regulation

Phenotype	Impaired-function	rs10830963	Non-carriers	Impaired-function vs Non-carriers		rs10830963 vs Non-carriers	
	Mean ± SE	Mean ± SE	Mean ± SE	Estimated effect (95% CI)	p	Estimated effect (95% CI)	p
$AUC_{glucose}$ (mmol/l x min)	140.3 ± 87.3	92.9 ± 22.9	22.8 ± 26.2	117.5 (-21.1, 256.0)	0.092	70.2 (-3.0, 143.4)	0.059
$iAUC_{glucose}$ (mmol/l x min)	69.2 ± 42.6	54.0 ± 12.1	4.9 ± 12.0	64.3 (-52.1, 180.7)	0.21	49.1 (13.8, 84.4)	0.0082
$AUC_{insulin}$ (pmol/l x min)	-14596.9 ± 8618.2	-14822.6 ± 8170.0	-5212.0 ± 3413.6	-9384.8 (-25213.0, 6443.3)	0.91	-9610.6 (-26556.7, 7335.6)	0.68
$iAUC_{insulin}$ (pmol/l x min)	-6436.3 ± 4287.6	-5498.0 ± 3851.4	-2270.1 ± 1579.7	-4166.2 (-11739.7, 3407.4)	0.27	-3227.9 (-11185.8, 4729.9)	0.68
AUC_{GIP} (pmol/l x min)	-1262.0 ± 214.8	-1309.0 ± 758.1	-1810.2 ± 692.5	548.2 (-977.9, 2074.2)	0.46	501.2 (-1608.5, 2610.9)	0.63
$iAUC_{GIP}$ (pmol/l x min)	-474.9 ± 321.0	-713.2 ± 414.3	-949.0 ± 378.4	474.1 (-892.3, 1840.5)	0.48	235.7 (-917.0, 1388.5)	0.68
AUC_{GLP-1} (pmol/l x min)	-116.9 ± 371.6	108.2 ± 219.7	84.4 ± 120.4	-201.3 (-817.8, 415.2)	0.59	23.8 (-465.4, 513.1)	0.92
$iAUC_{GLP-1}$ (pmol/l x min)	24.3 ± 221.6	120.9 ± 122.2	78.8 ± 73.7	-54.5 (-426.5, 317.5)	0.76	42.1 (-238.9, 323.1)	0.76
$AUC_{glucagon}$ (pmol/l x min)	-15.6 ± 48.3	-145.7 ± 93.7	-119.3 ± 80.6	103.7 (-92.2, 299.6)	0.28	-26.4 (-278.6, 225.9)	0.83
$iAUC_{glucagon}$ (pmol/l x min)	-26.0 ± 8.8	2.6 ± 18.8	14.2 ± 10.8	-40.2 (-79.2, -1.2)	0.044	-11.7 (-54.2, 30.9)	0.58
CIR	-283.5 ± 86.8	-316.0 ± 126.4	-56.6 ± 116.4	-226.9 (-615.4, 161.6)	0.24	-259.4 (-612.8, 94.0)	0.10
BIGTT-AIR	-432.1 ± 154.4	-978.4 ± 848.7	-480.2 ± 444.2	48.1 (-1500.0, 1596.3)	0.083	-498.2 (-2361.6, 1365.2)	0.88
$AUC30_{insulin}$ (pmol/l x min)	-3062.7 ± 810.6	-3006.1 ± 1081.8	-1533.8 ± 772.5	-1528.9 (-4216.6, 1158.8)	0.076	-1472.3 (-4133.6, 1188.9)	0.11
BIGTT-SI	1.0 ± 0.53	0.70 ± 0.49	0.43 ± 0.34	0.61 (-0.73, 1.9)	0.35	0.28 (-0.90, 1.5)	0.64
HOMA-IR	-1.4 ± 0.5	-1.7 ± 0.5	-0.2 ± 0.4	-1.2 (-2.7, 0.29)	0.07	-1.5 (-2.8, -0.24)	0.032

ESM Table 11 | Summary statistics of the phenotypes involved in glucose regulation. “Impaired-function” refers to carriers of the *MTNR1B* variants impairing MT2 signaling. “rs10830963” refers to homozygous carriers of rs10830963. “Non-carriers” refers to controls not carrying any of the examined *MTNR1B* variants. Abbreviations: iAUC, incremental AUC; GIP, glucose-dependent insulinotropic polypeptide; GLP-1, glucagon-like peptide-1; CIR, corrected insulin response; BIGTT-AIR, the beta cell function insulin sensitivity glucose tolerance test (BIGTT) – acute insulin response; AUC30, AUC at time 0–30 of an oral glucose tolerance test; BIGTT-SI, BIGTT – insulin sensitivity index; HOMA-IR, homeostatic model assessment for insulin resistance; SE, standard error; p, p-value.

ESM Fig. 1 | The positions of the *MTNR1B* variants in the MT2 structure

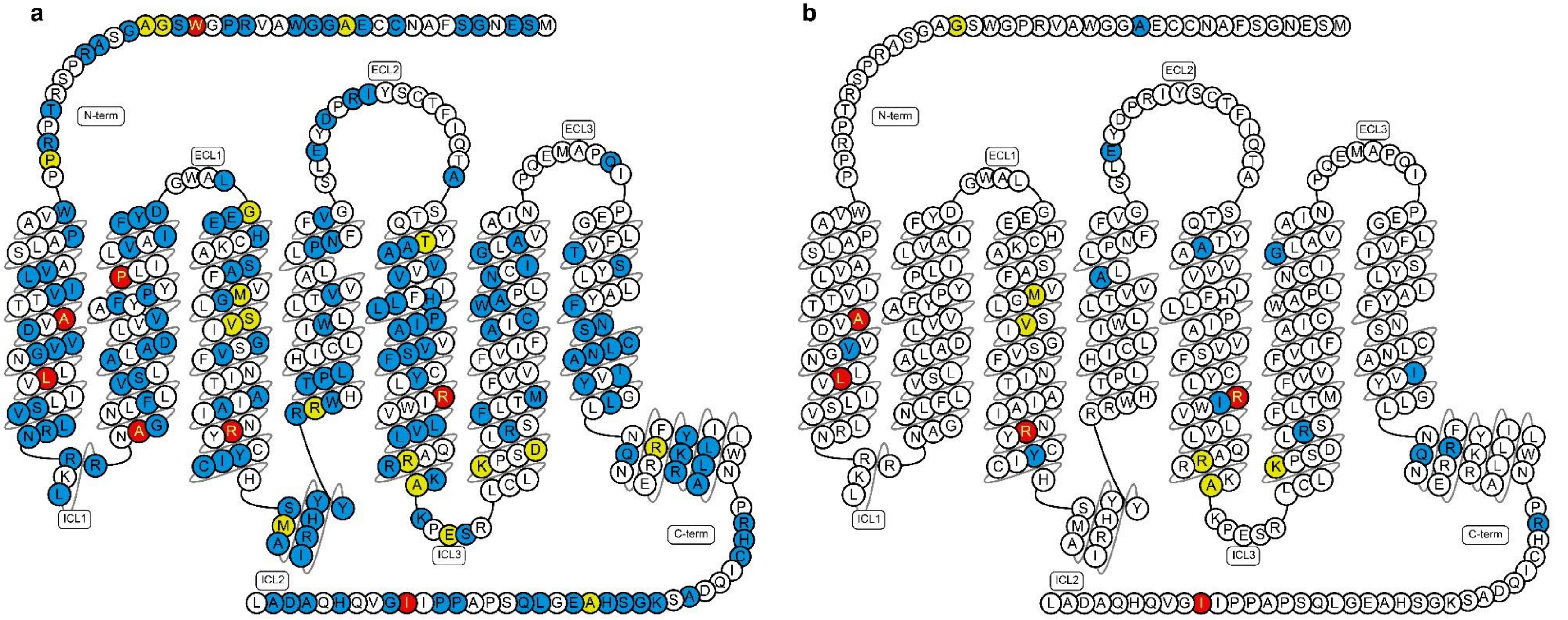

ESM Fig. 1 | Snakeplot of MT2 from GPCRdb [1], showing the structural positions of the nonsynonymous *MTNR1B* variants identified in the UK Biobank (**a**) and the Danish cohorts, Inter99 and DD2 (**b**). Coloring indicates whether a variant (represented in the given cohort) at the position was a WT-like variant (yellow) or an impaired-function variant (red) based on previous molecular characterization [2], otherwise non-tested variants (blue). Abbreviations: N-terminus (N-term), intracellular loop 1-3 (ICL1-3), extracellular loop 1-3 (ECL1-3), and C-terminus (C-term). In the UK Biobank, the nonsynonymous *MTNR1B* variants were distributed across the entire receptor structure, in which 8 of the 10 represented impaired-function variants were positioned in the transmembrane domains. In the Danish cohorts, most of the nonsynonymous *MTNR1B* variants were positioned in the transmembrane domains of the receptor structure, while a few were positioned in the extracellular N-terminal, the second extracellular loop, and the intracellular C-terminal. All represented impaired-function variants, except I353T, were also located in the transmembrane domains in the Danish cohorts.

ESM Fig. 2 | Burden testing of the common WT-like variant G24E and combining the impaired-function variants and pLoF variants in the UK Biobank

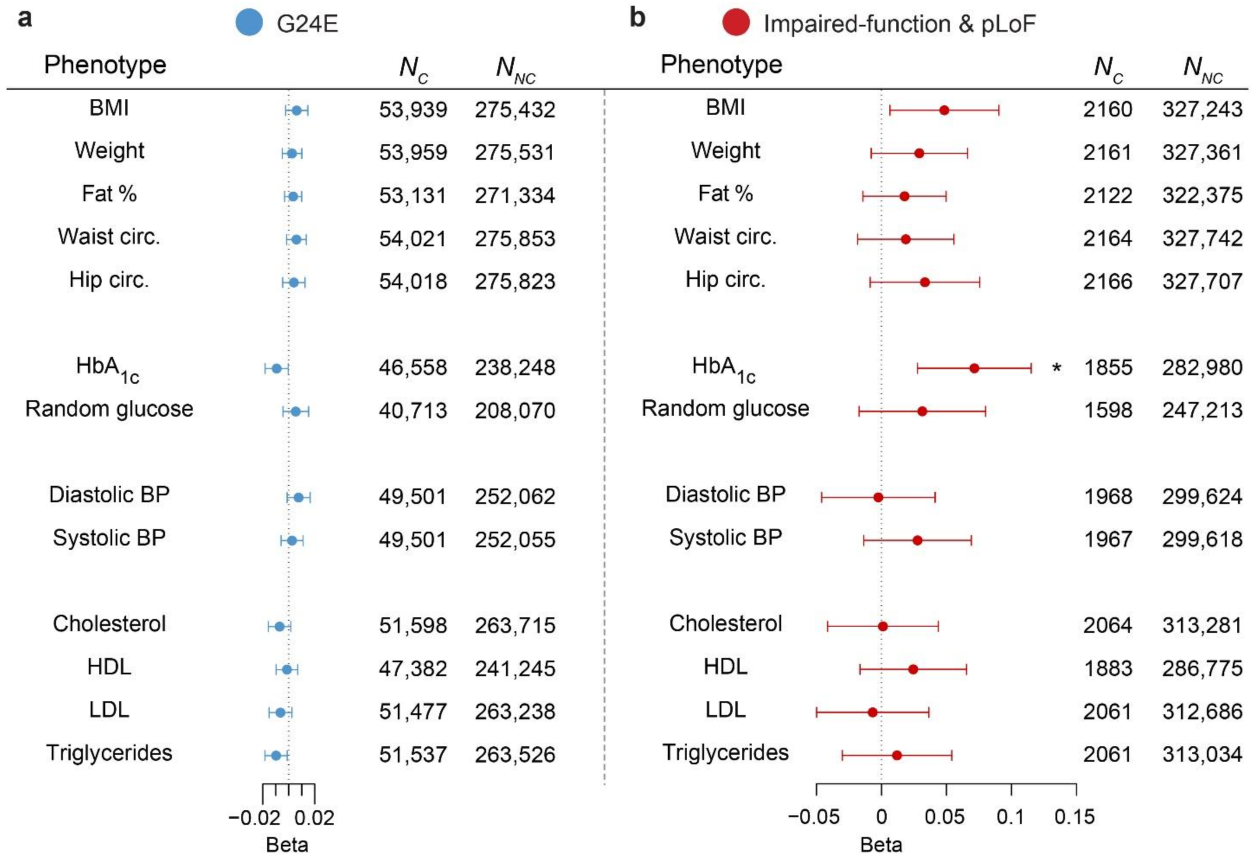

ESM Fig. 2 | The forest plots show the effect sizes as SD (beta with 95% CI) for the common G24E variant (blue, **a**) and the variant group combining impaired-function variants and pLoF variants (red, **b**), comprising 78 *MTNR1B* variants. N_C and N_{NC} represent the number of carriers and non-carriers, respectively. BMI, body mass index; circ., circumference; HbA_{1c}, glycated hemoglobin A_{1c}; BP, blood pressure; HDL, high-density lipoprotein; LDL, low-density lipoprotein. * Association reaching the corrected significance threshold ($p < 3.6 \times 10^{-3}$).

ESM Fig. 3 | Burden testing of the common WT-like variant G24E, and combined impaired-function variants and pLoF variants in the Danish population

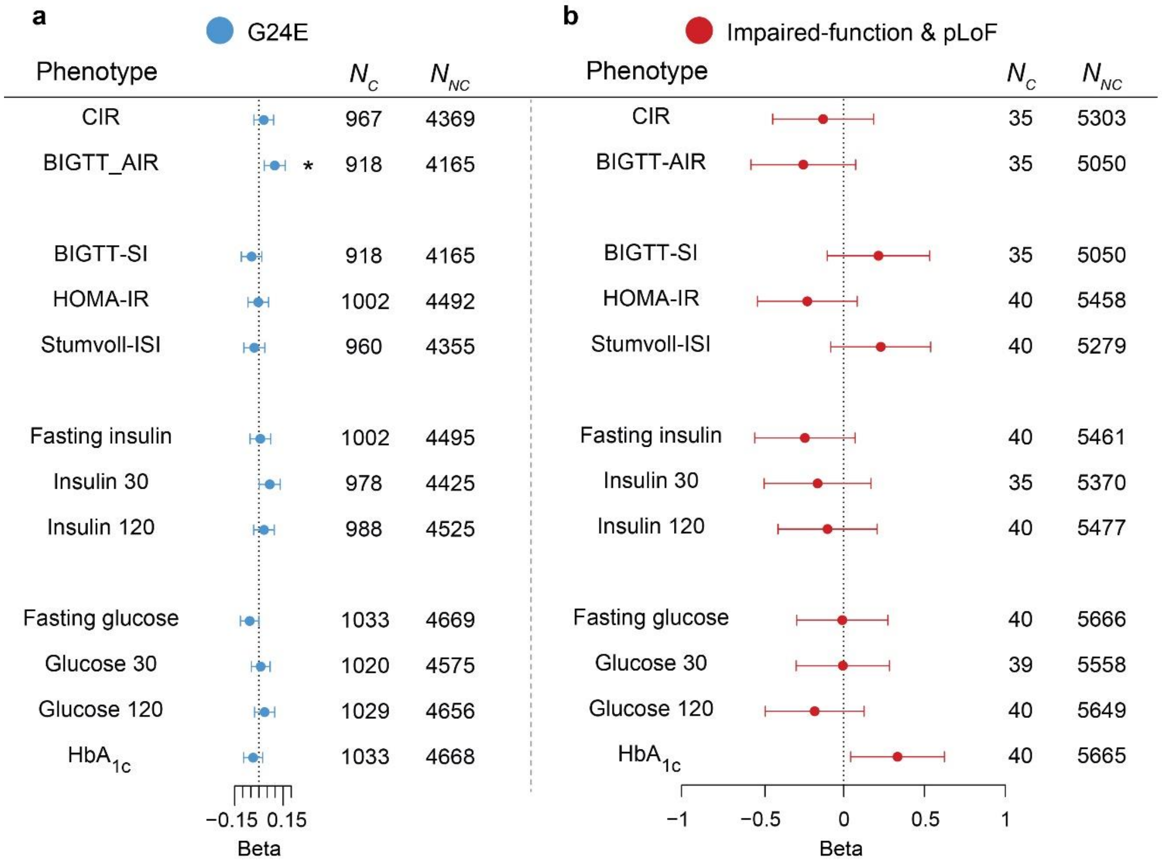

ESM Fig. 3 | The forest plots show the effect sizes as SD (beta with 95% CI) for the common G24E variant (blue, **a**) and the variant group combining impaired-function variants and pLoF variants (red, **b**), comprising 12 *MTNR1B* variants. N_C and N_{NC} represent the number of carriers and non-carriers, respectively. CIR, the corrected insulin response index; BIGTT-AIR, the beta cell function insulin sensitivity glucose tolerance test (BIGTT) – acute insulin response; BIGTT-SI, the BIGTT – insulin sensitivity index; HOMA-IR, the homeostasis model assessment of insulin resistance

index; Stumvoll-ISI, Stumvoll insulin sensitivity index; HbA_{1c}, glycated hemoglobin A_{1c}. Time points, 30 and 120, represent the minutes after initiation of an oral glucose tolerance test. * Association reaching the corrected significance threshold ($p < 3.8 \times 10^{-3}$).

ESM FIGURES REFERENCES

1. Pándy-Szekeres G, Munk C, Tsonkov TM, et al (2018) GPCRdb in 2018: adding GPCR structure models and ligands. Nucleic Acids Research 46(D1):D440–D446. https://doi.org/10.1093/nar/gkx1109

2. Bonnefond A, Clément N, Fawcett K, et al (2012) Rare MTNR1B variants impairing melatonin receptor 1B function contribute to type 2 diabetes. Nat Genet 44(3):297–301. https://doi.org/10.1038/ng.1053
